# Mitogen‐activated protein kinase activity drives cell trajectories in colorectal cancer

**DOI:** 10.15252/emmm.202114123

**Published:** 2021-08-19

**Authors:** Florian Uhlitz, Philip Bischoff, Stefan Peidli, Anja Sieber, Alexandra Trinks, Mareen Lüthen, Benedikt Obermayer, Eric Blanc, Yana Ruchiy, Thomas Sell, Soulafa Mamlouk, Roberto Arsie, Tzu‐Ting Wei, Kathleen Klotz‐Noack, Roland F Schwarz, Birgit Sawitzki, Carsten Kamphues, Dieter Beule, Markus Landthaler, Christine Sers, David Horst, Nils Blüthgen, Markus Morkel

**Affiliations:** ^1^ Institute of Pathology Charité – Universitätsmedizin Berlin, Corporate Member of Freie Universität Berlin and Humboldt‐Universität zu Berlin Berlin Germany; ^2^ IRI Life Sciences Humboldt University of Berlin Berlin Germany; ^3^ German Cancer Consortium (DKTK) Partner Site Berlin German Cancer Research Center (DKFZ) Heidelberg Germany; ^4^ BIH Bioportal Single Cells Berlin Institute of Health at Charité – Universitätsmedizin Berlin Berlin Germany; ^5^ Core Unit Bioinformatics (CUBI) Berlin Institute of Health at Charité Universitätsmedizin – Berlin Berlin Germany; ^6^ Max Delbrück Center for Molecular Medicine Berlin Institute for Medical Systems Biology (BIMSB) Berlin Germany; ^7^ Institute of Medical Immunology Charité – Universitätsmedizin Berlin, Corporate Member of Freie Universität Berlin and Humboldt‐Universität zu Berlin Berlin Germany; ^8^ BIFOLD – Berlin Institute for the Foundations of Learning and Data Berlin Germany; ^9^ Department of Surgery Charité – Universitätsmedizin Berlin, Corporate Member of Freie Universität Berlin and Humboldt‐Universität zu Berlin Berlin Germany

**Keywords:** cancer profiling, ERK, RNA velocity, single‐cell RNA sequencing, SLAM‐Seq, Cancer, Digestive System

## Abstract

In colorectal cancer, oncogenic mutations transform a hierarchically organized and homeostatic epithelium into invasive cancer tissue lacking visible organization. We sought to define transcriptional states of colorectal cancer cells and signals controlling their development by performing single‐cell transcriptome analysis of tumors and matched non‐cancerous tissues of twelve colorectal cancer patients. We defined patient‐overarching colorectal cancer cell clusters characterized by differential activities of oncogenic signaling pathways such as mitogen‐activated protein kinase and oncogenic traits such as replication stress. RNA metabolic labeling and assessment of RNA velocity in patient‐derived organoids revealed developmental trajectories of colorectal cancer cells organized along a mitogen‐activated protein kinase activity gradient. This was in contrast to normal colon organoid cells developing along graded Wnt activity. Experimental targeting of EGFR‐BRAF‐MEK in cancer organoids affected signaling and gene expression contingent on predictive KRAS/BRAF mutations and induced cell plasticity overriding default developmental trajectories. Our results highlight directional cancer cell development as a driver of non‐genetic cancer cell heterogeneity and re‐routing of trajectories as a response to targeted therapy.

## Graphical abstract

Colorectal cancer (CRC) cells can adopt a range of transcriptomic states. This study uses single cell RNA sequencing of primary CRC tissue and organoids to identify patient‐overarching CRC cell transcriptome clusters. RNA metabolic labelling indicates preferred CRC cell developmental trajectories.

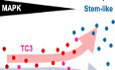

**The paper explained**

**Problem**

Cancer cells, like normal cells in our bodies, are thought to develop along preferred trajectories. No consensus exists how to define developmental states of colorectal cancer cells, and it is unknown which signals control their development.

**Results**

We define here patient‐overarching developmental states of colorectal cancer cells and find that the cancer cells develop along trajectories defined by activity of a signaling pathway termed MAPK. Targeting MAPK disturbed development of cultured colorectal cancer cells, sometimes resulting in cells heading toward an unwanted stem cell‐like state. This cellular behavior might explain cases of therapy resistance observed in patients.

**Impact**

Knowledge of developmental routes of single cancer cells is key for future improvement of targeted therapy.

## Introduction

Healthy cells in the human body develop along trajectories controlled by intrinsic and extrinsic signals to ensure tissue homeostasis. Cancer cells cannot maintain homeostasis, as oncogenic mutations activate signaling pathways cell‐intrinsically and render cancer cells unresponsive to paracrine signals (Hanahan & Weinberg, 2011). Colorectal cancer (CRC) commonly initiates via mutations activating Wnt/β‐catenin signaling that maintains stem cells in the normal colon epithelium (Fearon, 2011). Subsequent mutations deregulate further signaling pathways such as RAS‐RAF‐MEK‐ERK (also known as mitogen‐activated protein kinase; MAPK signaling) providing pro‐proliferative cues. Less frequently, CRC initiates via BRAF mutations or from chronic inflammation (Lasry *et al*, 2016; De Palma *et al*, 2019). Genetic CRC drivers have direct and indirect effects on cancer cell development and the cellular composition of CRC and its microenvironment.

There is substantial evidence for the existence of tumor cell subpopulations and clonal architecture in CRC depending on genetics, microenvironmental cues, and space constraints (Van Der Heijden *et al*, 2019). Cancer stem cells are defined by their capacity for self‐renewal and ability to initiate clonal outgrowth (Kreso & Dick, 2014). CRC cells with these characteristics have been distinguished by surface proteins like CD133, EPHB2, or LGR5 (O’Brien *et al*, 2007; Ricci‐Vitiani *et al*, 2007; Merlos‐Suárez *et al*, 2011). Likewise, lineage tracing in CRC cancer models has revealed preferential outgrowth of cancer cell subpopulations defined by expression of genes such as *LGR5* or *IL17RB* (Shimokawa *et al*, 2017; Goto *et al*, 2019) or by localization at the leading edge of the tumor (Lamprecht *et al*, 2017). Furthermore, CRC cells located at the invasive front and expressing genes such as the matrix metalloproteinase gene *MMP7* contributed disproportionally to metastasis (Brabletz *et al*, 1999; Vermeulen *et al*, 2010). While these studies suggest that CRC cells are heterogeneous and hierarchically organized, other studies stress that developmental capacities of CRC cells are subject to a high degree of plasticity. In particular, oncogenic mutations and paracrine signals have been shown to trigger reversal of developmental trajectories so that differentiated cells regain stem cell characteristics (Buczacki *et al*, 2013; Schwitalla *et al*, 2013; Jadhav *et al*, 2017).

Metastatic CRC is treated by chemotherapy and/or therapies targeting MAPK signaling, depending on predictive mutation status. Patients without RAS or RAF mutations profit from anti‐EGFR antibody therapy (Karapetis *et al*, 2008; Van Cutsem *et al*, 2009), while patients with BRAF‐mutant CRC now receive first‐line therapy combinations of anti‐EGFR antibodies and BRAF kinase inhibitors (Corcoran *et al*, 2018). Recent studies suggest roles for cell plasticity in therapy resistance. For instance, chemoresistance has been linked to subpopulations of CRC cells expressing the transcription factor ZEB2 (Francescangeli *et al*, 2020), and anti‐EGFR therapy resistance has been associated with rise of stem cell‐like populations (Lupo *et al*, 2020) and stromal remodeling (Woolston *et al*, 2019).

Taken together, emerging evidence suggests that hierarchically organized tumor cell heterogeneity and cell plasticity play key roles in CRC progression and therapy response. However, developmental states of CRC cells are not well‐defined, and it is not known whether transcriptome states are graded along preferential developmental trajectories. Here, we use single‐cell RNA sequencing to identify patient‐overarching CRC cell states defined by strengths of oncogenic signals and replicative responses. We use metabolic labeling of RNA in CRC organoids to delineate CRC development and show that CRC cell differentiation states, developmental trajectories, and therapy‐associated cell plasticity are informed by MAPK activity.

## Results

### CRC cells can assume patient‐overarching states

**Figure 1:**
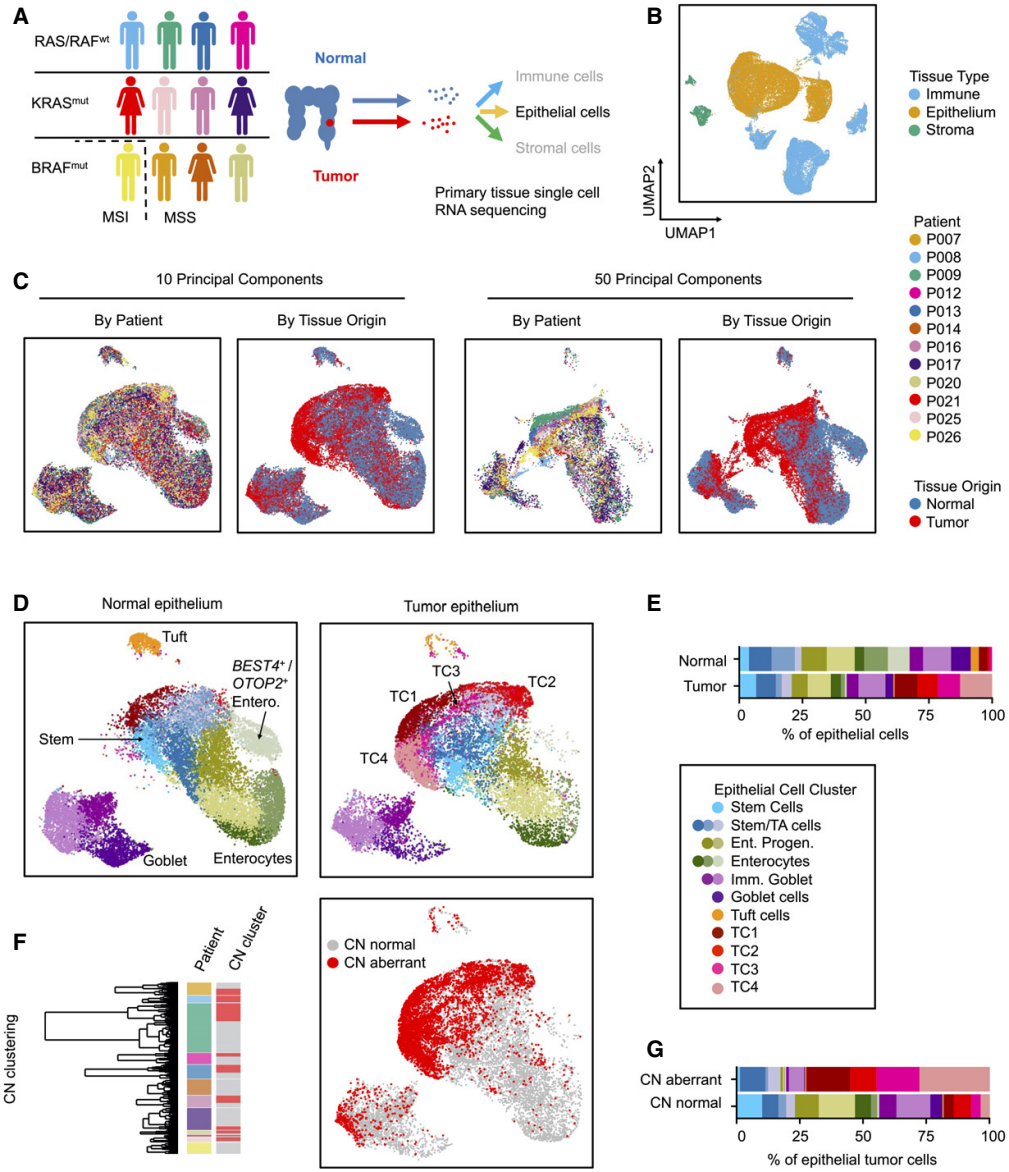
Generation of CRC single‐cell RNA sequencing data and epithelial cell type census AWorkflow of the Clinical Single Cell Sequencing pipeline. In short, CRC and adjacent non‐tumor tissue were sampled from 12 patients. Single‐cell RNA sequencing data were generated using the 10× Genomics platform, as outlined in Materials and Methods. For histology, see Appendix Fig S1. For patient characteristics, see Table EV1, for mutational data, see Table EV2.B–DUMAPs of single‐cell transcriptome data. (B) UMAPs of epithelial, immune, and stromal cell transcriptomes, color‐coded by tissue origin as assessed by marker genes. For marker genes, see Table EV3. (C) UMAPs of epithelial cell transcriptomes, color‐coded by patient identity or tissue of origin, as indicated. (D) UMAPs of epithelial cells, separated by tissue of origin (normal vs. tumor). Clusters are color‐coded by cell identity, as inferred from marker genes as outlined in main text. For epithelial cell cluster marker genes, see Table EV4.ERelative fractions of epithelial cell states for all patients.FIdentification of copy number‐aberrant versus normal epithelial cells in tumor tissue. To the left: Cell cluster dendrogram, color‐coded by patient and by copy number‐associated clusters (*n* = 2 per patient, copy number normal cluster: gray; copy number‐aberrant cluster: red). To the right: Localization of SCN‐aberrant cells in the UMAP (red).GRelative fractions of epithelial cell states for SCN‐normal versus SCN‐aberrant cells for all patients. For fractions by patient and copy number status, see Fig EV2.

To capture the diversity of CRC cell states compared to the normal colon epithelium, we performed single‐cell transcriptome analysis of twelve previously untreated CRC patients undergoing primary surgery (Fig 1A). We utilized tissue samples that included the invasive tumor front and matched non‐cancerous tissues (Appendix Fig S1). Tumors encompassed stages pTis (Tumor *in situ*) to pT4, with or without metastasis, and with various locations along the cephalocaudal axis of the colon (Table EV1). Genetic analysis revealed mutational patterns characteristic for canonical CRC progression in most tumors; however, tumors from patients P007, P014, P020, and P026 contained the BRAF^V600E^ mutation often associated with the serrated progression pathway and tumor P008 was colitis‐associated (Tables EV1 and EV2). Eleven patients were diagnosed with microsatellite‐stable (MSS) CRC, while the tumor of patient P026 was microsatellite‐instable (MSI). We produced transcriptome libraries using a commercial droplet‐based system and sequenced the libraries to obtain transcriptomes covering 500–5,000 genes per cell. Transcriptomes were clustered, and clusters were allocated to epithelial, immune, or stromal subsets, using known marker genes (Smillie *et al*, 2019) (Fig 1B, Appendix Fig S2, Table EV3), and more than 30,000 epithelial cell transcriptomes were analyzed further.

Normal and tumor‐derived epithelial cell transcriptomes of all patients largely intermingled when visualized in a common Uniform Manifold Approximation Projection (UMAP) employing ten principal components (McInnes *et al*, 2018), but partially separated when using a higher number of 50 components for UMAP embedding (Fig 1C). Separation by patient occurred particularly in areas enriched for tumor‐derived transcriptomes suggesting the existence of patient‐specific gene expression in cancer epithelium. In summary, the UMAP embedding indicates that our single‐cell data are largely free from sample‐specific bias, but instead reflect intrinsic differences between normal and tumor cell transcriptomes.

We used cell type‐specific signatures and marker genes to annotate the epithelial cell clusters (Smillie *et al*, 2019; Fig 1D and E; Tables EV3 and EV4). In the normal epithelium, we identified stem cells by markers such as *LGR5* and *OLFM4*. Neighboring clusters were annotated as enterocyte progenitors or mature enterocytes by expression of absorptive lineage markers including *KRT20* and *FABP1* (Appendix Fig S3, Table EV4). *BEST4*‐ and *OTOP2*‐expressing enterocytes formed a discrete cluster (Parikh *et al*, 2019). Further separate epithelial clusters were identified as immature and mature secretory goblet cells expressing *MUC2* and *TFF3,* and as tuft cells expressing *TRPM5*. Four clusters formed largely from tumor cell transcriptomes, termed TC1‐TC4. These clusters were defined by high, but also unequal, levels of stem cell markers such as *OLFM4*, *CD44,* and *EPHB2* and DNA repair genes such as *XRCC2*. *MMP7* was among the few genes expressed exclusively in cancer, but not in the normal epithelium (Appendix Fig S3). Clusters populated by differentiated absorptive and secretory cells were reduced in tumors, and profiles representing tuft cells and *BEST4*/*OTOP2*‐positive enterocytes were vastly underrepresented.

**Figure EV1:**
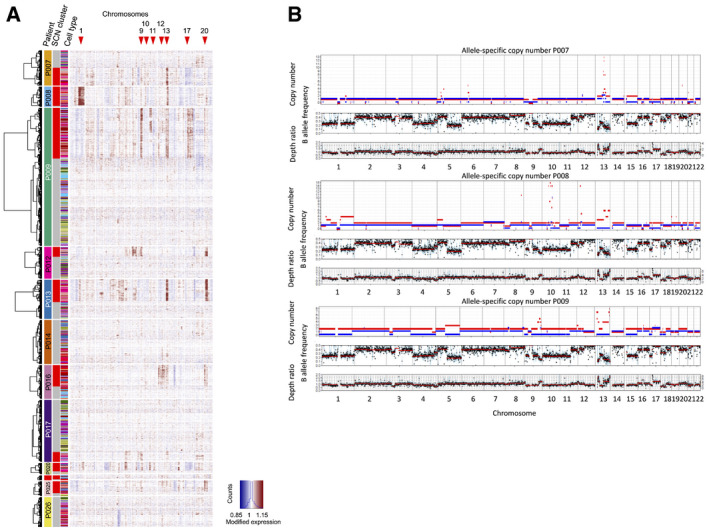
Assessment of copy number variations Copy number calling from single‐cell transcriptomes, using InferCNV.Copy number calling from exome sequences. To validate SCNAs from single‐cell transcriptome data, we performed allele‐specific SCNA calling for patients P007, P008, and P009 from bulk whole exome data. Germline variants were discovered *de novo,* and read counts were accumulated for each allele at heterozygous germline variants using bcftools v1.9 multi‐allelic caller. Discovered variants and read counts were passed to Sequenza 3.0 for segmentation and calling of allele‐specific SCNAs. Top tracks: copy number segments of major and minor alleles. Middle tracks: B (minor) allele frequency. Bottom tracks: depth ratio of matched tumor versus normal samples with corresponding second axis of inferred copy number. P007 showed multiple LOH events on chromosomes 1, 3, 5, 6, 8, 9, 13, 15, 17, and 21 as well as a focal amplification on chromosome 13. P008 demonstrated a similar SCNA pattern with LOH events on chromosomes 8, 9, 10, 12, 13, 17, and 22 as well as multiple focal amplifications on chromosomes 8, 10, 12, and 13. P009 was characterized by widespread copy number neutral LOH in about 50% of the genome. Focal amplifications were found on chromosomes 9 and 13.

Microsatellite‐stable CRC is defined by somatic copy number aberrations (SCNAs). Thus, we next distinguished cancer from normal epithelial transcriptomes derived from the tumor tissues by inferring SCNAs. We identified clusters of SCN‐aberrant epithelial cells in ten out of twelve tumors (Figs 1F and Fig EV1A). Exome sequencing of tumors P007, P008, and P009 validated SCNA calling from transcriptomes, showing that the procedure is robust for our single‐cell data (Fig EV1B). P014 and P026 contained no cells with overt SCNAs. This was expected for tumor P026, which is MSI, but unexpected for P014, which was diagnosed as BRAF‐mutant, however MSS. In‐depth analysis of patient‐specific gene expression patterns (Appendix Fig S4A) and protein distributions (Appendix Fig S4B) revealed that patient‐specific differences in cancer cell transcriptomes are at least partly driven by individual patterns of genomic gains and losses (Appendix Fig S4C–E).

**Figure EV2:**
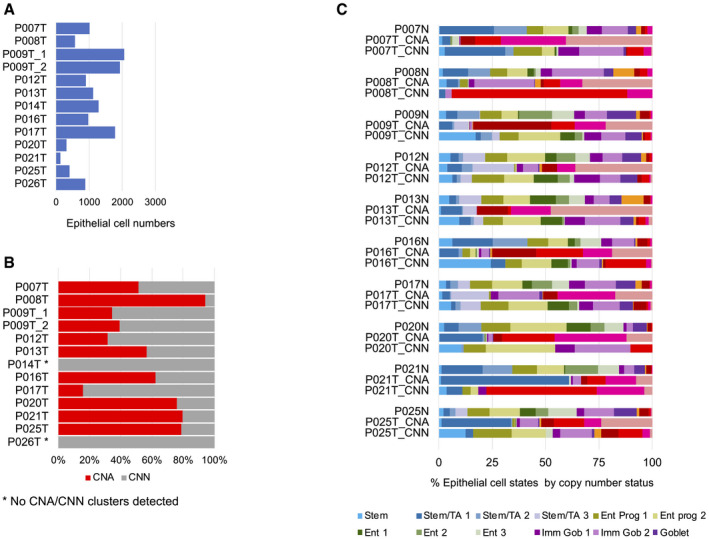
Cell type census, per patient Epithelial cell numbers per tumor sample retrieved by our analysis pipeline.Copy number calling on tumor samples. No copy number clusters could be called for P014T and P026T (see also Fig EV1A).Relative prevalence of cell differentiation states in the epithelial compartment, per patient. Cell state prevalence from tumor samples is given for copy number‐aberrant (CNA) versus copy number normal (CNN) cells.

More than 86% of the TC1‐4 cells were called SCN‐aberrant, along with substantial fractions of cells defined as stem/transient‐amplifying (TA)‐cell‐like (36%) or immature goblet cells (22%, Fig 1F and G, and Fig EV2 for data by patient, Table EV5). In contrast, a large majority (95%) of mature absorptive enterocytes and mature goblet cells derived from tumor samples were identified as copy number normal and therefore likely stem from non‐cancerous tissue at the tumor margins. In summary, our analyses define six main patient‐overarching clusters of CRC cells: CRC cells resembling normal stem/TA‐cell‐like cells, CRC cells resembling immature goblet‐like cells, and CRC cells in the TC1‐4 clusters with transcriptomes distinct from well‐defined cell types of the normal colon epithelium.

### Epithelial tumor cell clusters differ by oncogenic traits and signals

**Figure 2:**
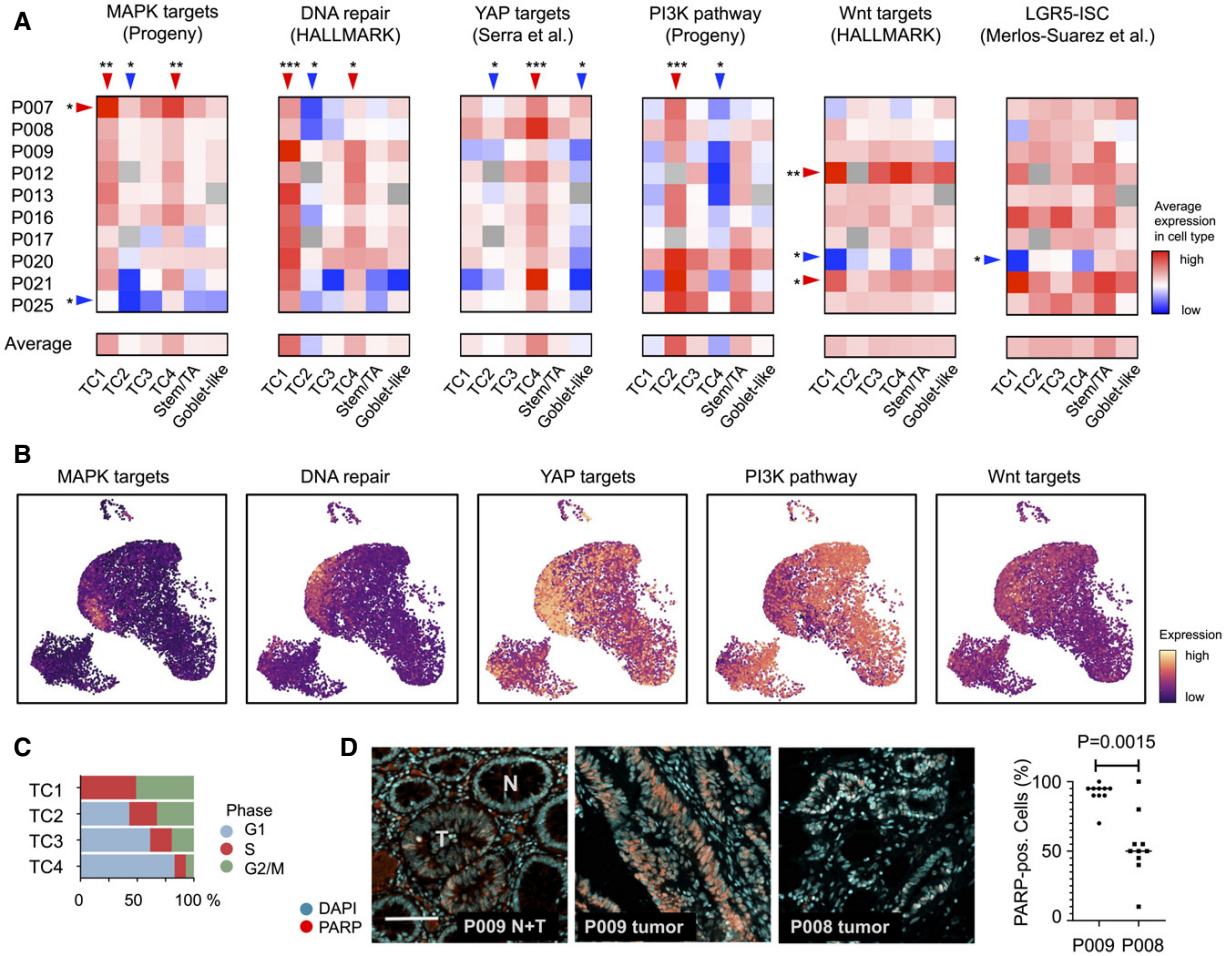
CRC cell clusters are distinguished by signaling pathway activities Transcriptional activity associated with key oncogenic traits and signals, by tumor‐specific cell type and patient, as indicated. Red: high activity, blue: low activity. Significance was assessed by Kruskal–Wallis test (FDR‐corrected *P* < 0.05), followed by a post hoc analysis using a Wilcox test of each group against all other groups, FDR‐corrected significance levels are shown (*P < 0.05; **P < 0.01; ***P < 0.001).Visualization of signatures corresponding to oncogenic traits and signals in the tumor cell transcriptome UMAP.Cell cycle distribution of TC1‐4 epithelial tumor cells, as inferred from single‐cell transcriptomes.Immunofluorescence of DNA damage‐associated nuclear protein PARP. Images show adjacent normal and tumor crypts of tissue P009T, marked by N and T, respectively, and tumor tissue of patients P008 and P009, as indicated. Scale bar 100 µm. Significance was assessed by an unpaired t‐test, after blinded analysis of 10 random images per tumor.

We next defined characteristics of the six CRC cell clusters by computing relative strengths of transcriptional footprints related to oncogenic signaling and cancer‐associated functional traits, using transcriptome data of all copy number‐aberrant cells (Fig 2A and B). TC1 and TC4 were significantly enriched for the expression of direct MAPK/ERK targets (*P* = 0.004 and *P* = 0.006, FDR‐corrected post hoc Wilcox tests, respectively; Schubert *et al*, 2018), across all tumors and in particular in P007. We furthermore found a strong association of TC1 cells with the expression of hallmark signatures related to DNA repair and the G2/M replication checkpoint across all individual cancers (*P* = 1.4E‐10; Liberzon *et al*, 2015). This indicates that TC1 cluster cancer cells experience high levels of replication stress, a therapy‐relevant trait of many cancers, including CRC. Indeed, TC1 cluster cells were exclusively assigned to the S or G2/M cell cycle phases by gene expression (Fig 2C), in line with cells under replication stress, as also seen by *XRCC2* expression (Appendix Fig S3). The DNA damage‐associated protein PARP stained many nuclei of the TC1‐high CRC tissue P009 but not of TC1‐low P008 (Fig 2D). Cancer cells clustered in TC4 were characterized by expression of an intestinal YAP target signature (*P* = 2.2E‐8; Serra *et al*, 2019). YAP transcriptional activity is linked to regenerative responses and tumor progression (Zanconato *et al*, 2016). TC2 transcriptomes were significantly associated with high PI3K pathway activity (*P* = 8.9E‐10), related to control of metabolism and apoptosis. Wnt/β‐catenin target gene activity was high across all TC clusters, but stem/TA‐cell‐like cancer cells showed stronger expression of a LGR5‐ISC stem cell signature that is Wnt‐driven (Merlos‐Suárez *et al*, 2011; Muñoz *et al*, 2012), but this association was not significant across the patients. In summary, assessment of cell signaling signatures provides information on pathway activities of epithelial cancer cell clusters and specific features of individual tumors. The analyses indicate that assignment of cancer cell transcriptomes to the TC1‐4 clusters reflects, at least partially, differential states of oncogenic networks and oncogene‐induced functional traits.

**Figure EV3:**
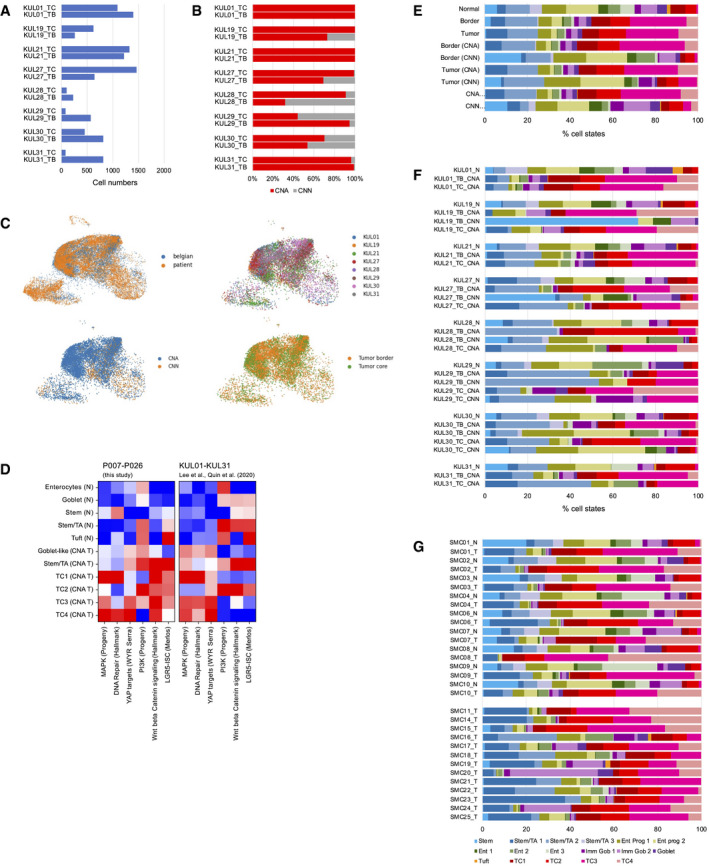
Analysis of scRNA‐seq data from validation cohorts A–FAnalysis of the KUL01‐KUL31 cohort (Lee *et al*, 2020; Qian *et al*, 2020). (A) Epithelial cell numbers per sample retrieved by our analysis pipeline. (B) Copy number calling on tumor border (TB) and tumor center (TC) samples. (C) Distribution of KUL01‐KUM31 transcriptomes on the reference UMAP. Top left: blue: KUL01‐KUL31 transcriptomes. Orange: Transcriptomes from P007‐P026 samples; top right: Distribution of KUL01‐KUM31 transcriptomes, by patient; bottom left: distribution of copy number normal versus aberrant transcriptomes; bottom right: distribution of tumor border versus tumor center transcriptomes. (D) Signature strengths of transcriptome clusters from our study (P007‐P026), compared to KUL01‐KUL31 transcriptome clusters after imputation. (E) Cell state distribution in the KUL01‐KUL31 cohort, by category. (F) Cell state distribution in the KUL01‐KUL31 cohort, by sample.GCell state distribution in the SMC01‐SMC25 cohort (Lee *et al*, 2020), by sample.

We validated our model of six patient‐overarching CRC cell states using single‐cell data from Belgian and Korean patient cohorts (Lee *et al*, 2020; Qian *et al*, 2020). We could confirm a prevalence of TC1‐4, stem/TA‐cell‐like and goblet‐cell‐like transcriptomes in SCN‐aberrant cancer cells compared to SCN‐normal and normal tissue epithelial cells and verified differential signaling pathway activities between the clusters (Fig EV3).

### CRC developmental trajectories follow a MAPK gradient

**Figure EV4:**
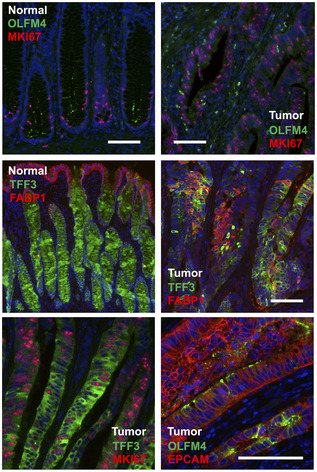
Assessment of CRC cell heterogeneity by immunofluorescence Immunofluorescence analysis for OLFM4, MKI67, FABP1, and TFF3 in normal and tumor tissue. All sections are from patient P009, except the EPCAM/OLFM4 co‐staining that was done on tumor tissue of P016. Scale bars indicate 100 µm. For marker expression in UMAP space, see Appendix Fig S3.

**Figure 3:**
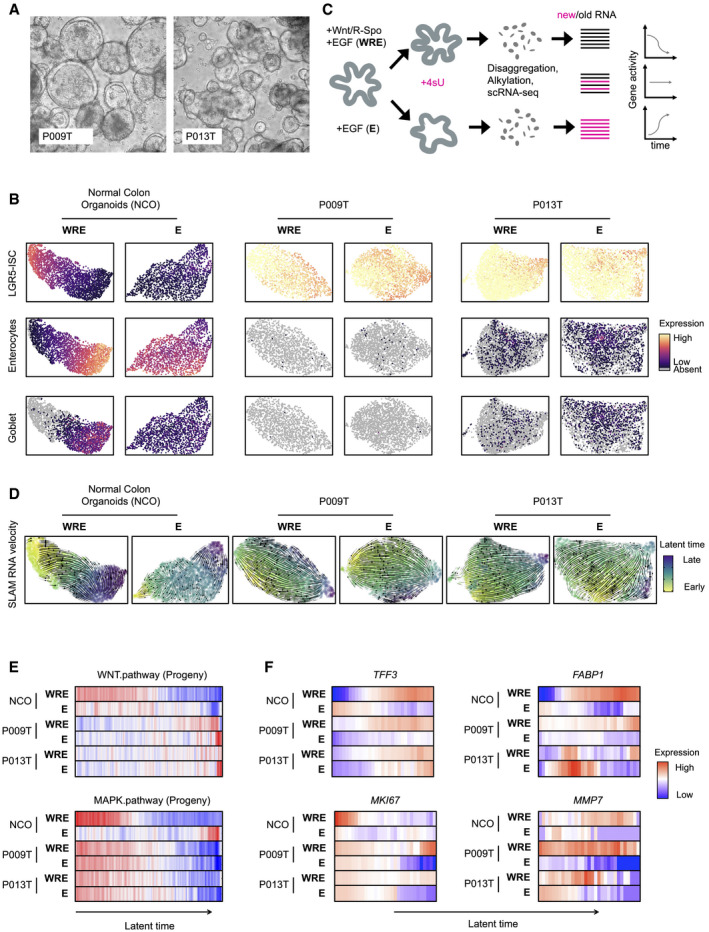
RNA metabolic labeling defines tumor cell trajectories in patient‐derived organoids Phenotypes of patient‐derived organoid lines P009T and P013T.UMAPs of organoid single‐cell transcriptomes. Organoid lines and medium conditions as indicated. LGR5‐ISC stem cell, enterocyte, and Goblet cell signatures are visualized.Schematic representation of workflow to infer RNA dynamics (“RNA velocity”). In short, organoids were passaged and assigned to different medium conditions. After three days, nascent RNA was metabolically labeled for 2 h using 4sU. Organoids were harvested, dissociated, and fixed. RNA in single cells was alkylated, and cells were subjected to single‐cell sequencing. Reads were assigned to nascent or old RNA status, depending on diagnostic T‐C mutational status.Developmental trajectories inferred from RNA metabolic labeling. Bold and thin arrows indicate high versus low directionality of RNA velocity. Colors below RNA velocity show latent time (yellow: early latent time; blue: late latent time).Activities of Wnt/β‐catenin and MAPK target genes in organoid single‐cell transcriptomes, ordered along latent time.Activities of *TFF3*, *FABP1*, *MKI67,* and *MMP7* in organoid single‐cell transcriptomes, ordered along latent time. Data information: Color code for panels E and F: Red: high activity; blue: low activity.

Immunofluorescent staining of primary CRC sections with antibodies directed against the stem cell marker OLFM4, the proliferation marker KI67, and the differentiation markers TFF3 and FABP1 revealed cell heterogeneity, but not how cancer cells in the tissue are related to each other (Fig EV4). To establish whether CRC cells are hierarchically organized, we established organoid lines of two tumor samples, P009 and P013 (Fig 3A). Organoids matched the cancer tissue on a mutational level (Table EV2). We cultured the cancer organoids, termed P009T and P013T, as well as normal colon organoids, termed NCO, in medium containing Wnt, R‐Spondin, and EGF (WRE medium) or alternatively in medium lacking Wnt and R‐Spondin (E medium). NCO organoids cultured in WRE medium showed graded expression of the intestinal LGR5‐ISC stem cell signature, while expression of differentiation markers was graded in the opposite direction (Fig 3B). LGR5‐ISC signature activity was lost when NCO organoids were cultured in E medium. In P009T and P013T CRC organoids, LGR5‐ISC signature activity was higher and independent of Wnt/R‐Spondin, while expression of differentiation signature genes was much lower. Taken together, these expression patterns are in line with Wnt‐dependent stem cell maintenance in normal tissue, and Wnt‐independent stem cell maintenance and block of terminal differentiation in cancer tissue with APC mutations. The data however do not show whether graded developmental trajectories exist in CRC.

We, therefore, metabolically labeled RNAs of the organoids by 4‐thio‐uridine (4sU), before dissociation and single‐cell sequencing (scSLAM‐Seq; Fig 3C; Herzog *et al*, 2017; Jürges *et al*, 2018). This allowed us to distinguish nascent labeled from older non‐labeled mRNA, to order cells along inferred latent time based on dynamic RNA expression (Bergen *et al*, 2020), also known as RNA velocity (Appendix Fig S5 for quality controls). When cultured in WRE medium, developmental trajectories of normal NCO organoids initiated in areas of maximal LGR5‐ISC signature scores and terminated in a region containing differentiated cells (Fig 3D). When cultured without Wnt/R‐Spondin in E medium, NCO normal colon organoids lost uniform direction of RNA velocity. In contrast, the P009T and P013T cancer organoids maintained strong transcriptional trajectories regardless of Wnt/R‐Spondin in the medium.

We ordered organoid transcriptomes along latent time and assessed strengths of oncogenic signals (Fig 3E). In line with the key role of Wnt in stem cell maintenance, normal colon organoids showed a gradient of Wnt/β‐catenin target gene expression along latent time when cultured in WRE medium. In contrast, both P009T and P013T cancer organoids showed no graded Wnt/β‐catenin‐related expression, but a clear gradient of MAPK target gene activity along latent time in both medium conditions. *TFF3*, marking secretory differentiation, was graded in CRC organoids along latent time, but *FABP1*, marking absorptive differentiation in the normal colon, was not (Fig 3F), in line with *TFF3* marking differentiated goblet‐like CRC cells at the end of developmental trajectories. Proliferation marker *MKI67* was confined to the beginning of the latent time trajectory of normal organoids in WRE medium and showed extended gradients in CRC organoids. Both P009T and P013T organoids displayed Wnt‐dependent loss of *MKI67* expression along latent time and also Wnt‐dependent *MMP7* expression. In summary, our metabolic RNA labeling experiments indicate decreasing MAPK activity along CRC developmental trajectories and suggest a role for Wnt as a paracrine signal influencing gene expression, such as *MMP7*, in APC‐deficient CRC cells.

### MAPK target gene expression defines CRC differentiation states

**Figure 4:**
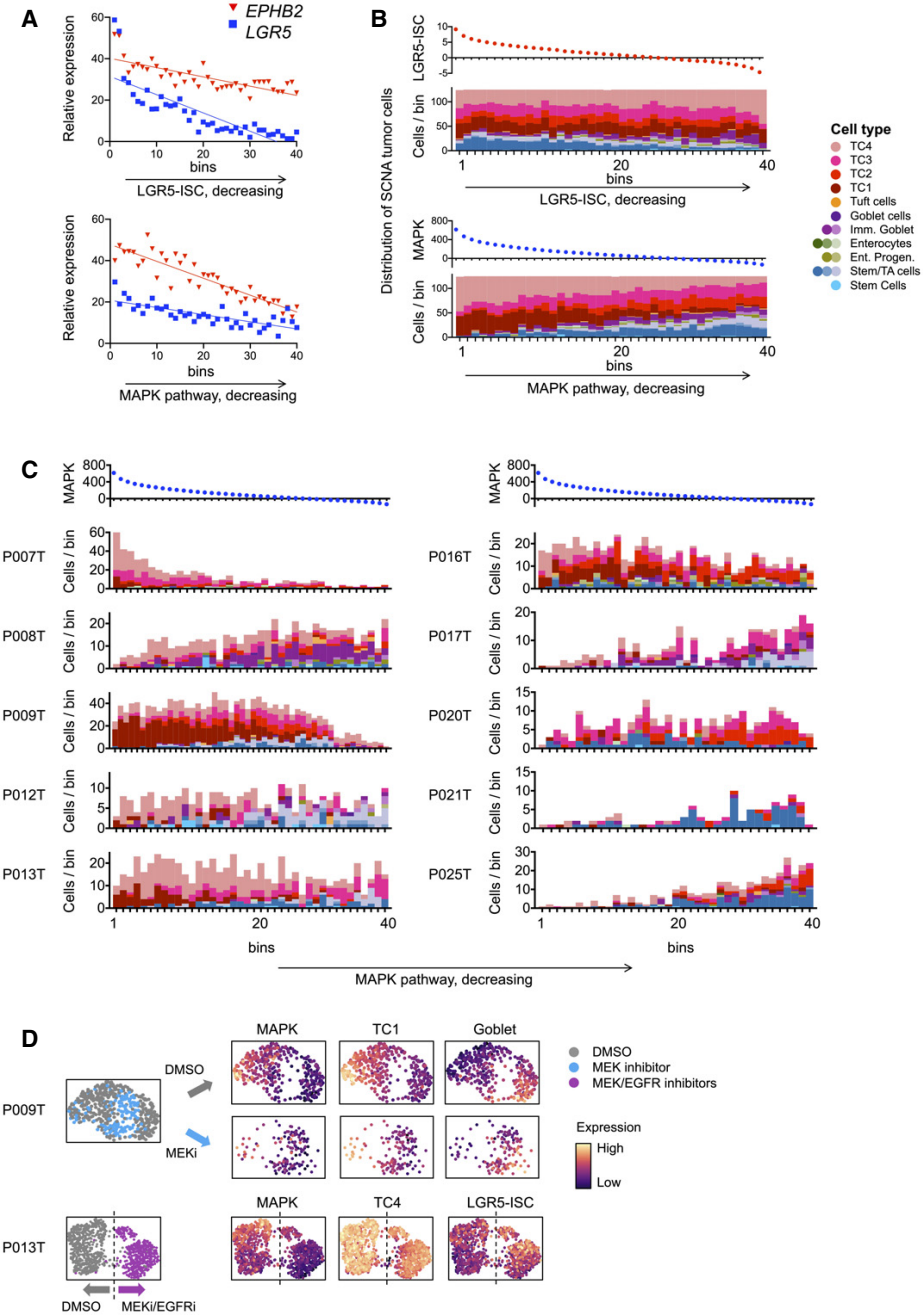
MAPK activity is linked to CRC cell differentiation states Gene expression of *LGR5* and *EPHB2*, along activity gradients of LGR5‐ISC or MAPK target gene signatures.Cell state distribution of SCN‐aberrant CRC cells along gradients of LGR5‐ISC or MAPK transcriptional signatures, as in A.Cell state distribution of SCN‐aberrant CRC tumor cells along MAPK signature activity, as in B, per tumor. Correlation between cell state distributions and MAPK target gene was calculated using Pearson’s r. For correlations and significances, see Table EV6. Color code as in Fig 4B.UMAP representations of single‐cell transcriptomes derived from P009T or P013T organoids, after MAPK blockade using MEK or combined MEK and EGFR inhibition. Color codes are treatment conditions or expression strength of signature, as indicated. Dashed line in P013T UMAP roughly separates control (DMSO) and MEK/EGFR inhibitor‐treated cells.

As MAPK‐related gene expression was associated with developmental trajectories in CRC organoids, we analyzed whether the previously defined states of primary CRC cells were organized along a MAPK gradient in primary CRC. We assigned the SCN‐aberrant primary cancer cell transcriptomes to 40 bins along a gradient of diminishing LGR5‐ISC gene signature activity or along decreasing MAPK activity, and determined expression levels of the stem cell markers *LGR5* and *EPHB2* (Merlos‐Suárez *et al*, 2011; Fig 4A). As expected, ordering transcriptomes by LGR5‐ISC signature strength resulted in graded expression of *LGR5* and *EPHB2*, and the signature was especially suited for aggregating cells with high *LGR5* expression. However, MAPK target gene signature strength performed even better in sorting cells along a gradient of *EPHB2* expression. We conclude that expression patterns of known CRC cell hierarchy markers agree with MAPK‐driven development.

When ordering primary CRC transcriptomes along a gradient of LGR5‐ISC activity, a higher proportion of stem/TA‐like tumor cells aggregated at the high end of the gradient, whereas tumor cells assigned as immature goblet cell‐like accumulated in the lower end, and TC1‐4 cells displayed a broad distribution (Fig 4B). In contrast, ordering of CRC cells by MAPK signature activity significantly enriched TC1 and TC4 cells at the start of the gradient (*P* = 8.6E‐23 and *P* = 7.6E‐20, respectively, adjusted Pearson’s chi‐squared p‐value), whereas stem/TA‐ and immature goblet cell‐like cells concentrated at the lower end (*P* = 1.3E‐19 and *P* = 1.1E‐8, respectively), and these aggregate differences were also preserved as graded cell state distributions along the MAPK axis in individual patients (Fig 4C, Table EV6): P007 showed the highest MAPK activity, and the highest proportion of TC4 cells which clustered in the MAPK‐high bins. TC1 and TC4 cells had also the highest average MAPK activities in most other CRCs, including P008, P012, P013, and P016. In contrast, stem‐/TA‐like tumor cells and immature goblet‐like CRC cells displayed relatively low expression of MAPK targets in all tumors, particularly in P009, P012, P013 P017, P021, P025, and P008, P017, respectively.

To experimentally determine whether the CRC cell states are functionally linked to MAPK activity, we blocked MAPK signaling in CRC organoids by the MEK1/2 inhibitor Selumetinib (AZD6244) or by Selumetinib in combination with the EGFR inhibitor Sapatinib (AZD8931) and analyzed singe‐cell gene expression after 48 h (Fig 4D). We found that cells showing a high TC1 signature were diminished after MEK inhibition in P009T organoids, while the fraction of cells with a high goblet‐cell‐like signature was increased. Likewise, P013T organoid cells showed lower expression of the TC4 signature genes after MAPK blockade and higher expression of the stem cell‐related LGR5‐ISC signature. These results suggest that CRC cell states are MAPK‐driven.

### Targeted therapy alters cell signaling networks in CRC organoids

**Figure 5:**
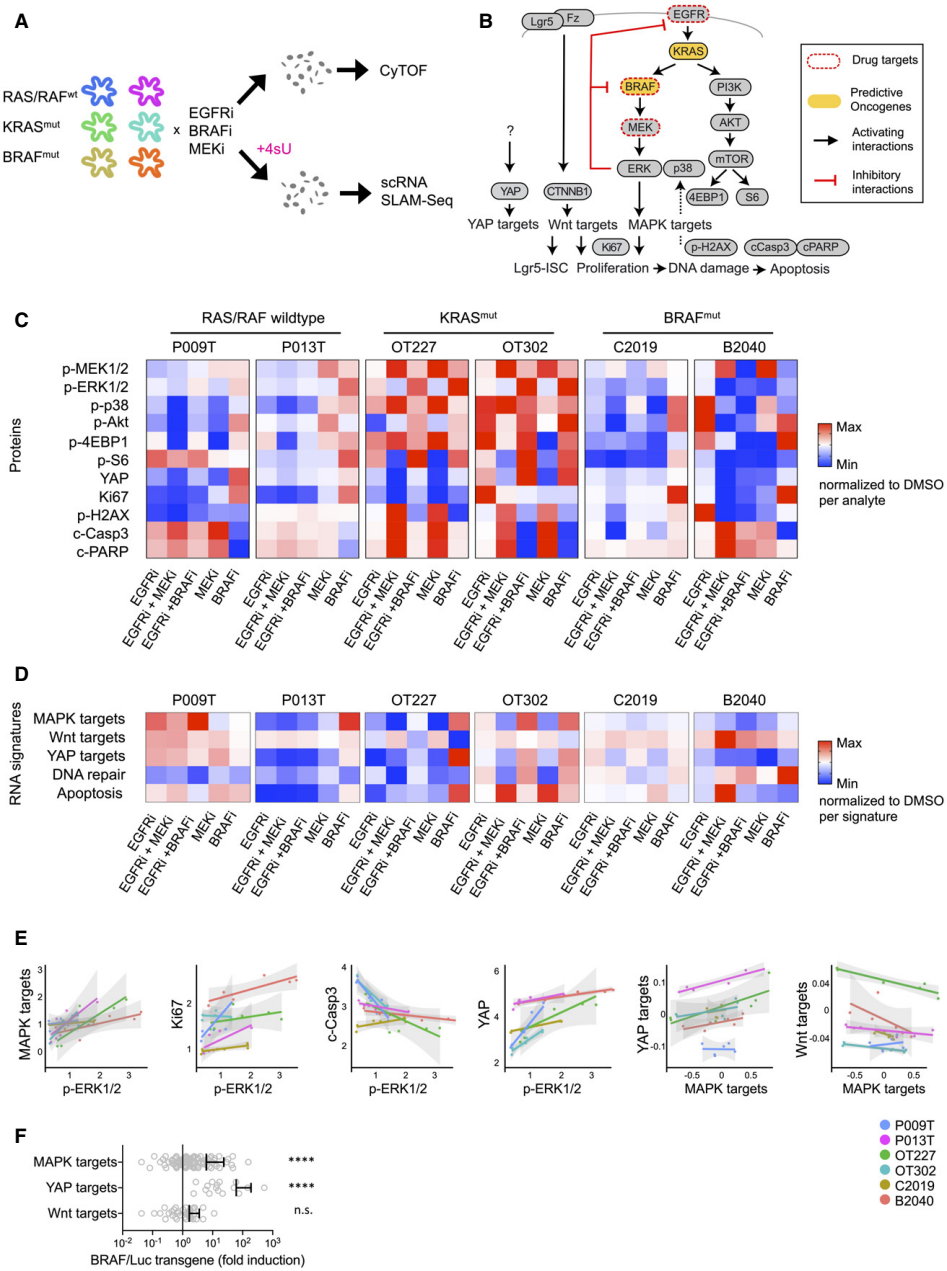
Anti‐MAPK therapy affects signaling networks and transcriptomes contingent on predictive mutations in organoids Workflow of the experimental therapy experiment. In short, organoids were treated for 48 h with inhibitors before disaggregation into single cells for CyTOF and scRNA‐seq analysis. For scRNA‐seq, organoids were labeled for 2 h with 4sU.Schematic representation of signaling network and transcriptional signatures associated with phenotypes or signaling pathways, as indicated.Heatmap of key CyTOF data. Average activities of selected analytes are given as log fold change after normalization to DMSO control condition. Range of color scale was adjusted for each analyte. For relative changes between all analytes, see Appendix Fig S7).Heatmap of signature gene expression, from scRNA‐seq data. Average activities of selected gene signatures are given as log fold change after normalization to DMSO control condition. Range of color scale was adjusted for each signature. DNA repair and Apoptosis are Broad Institute Hallmark signatures.Correlations between and within the CyTOF and scRNA‐seq datasets. For each line, average protein analyte (CyTOF) or transcriptional signature (scRNA‐seq) values were plotted for all six experimental conditions. Graphs give trend line and confidence intervals.Upregulation of MAPK and YAP target genes in mouse intestinal organoids after induction of oncogenic BRAF. Wnt target genes are not significantly affected. For experimental details, see Riemer *et al* (2015). Graph displays data points for signature gene expression values, mean and standard deviation. ****indicates *P*‐value of < 0.0001 in two‐tailed paired *t*‐test of gene expression ratios (luciferase control versus BRAF‐induced). n.s.: not significant.

MAPK is a key target of CRC therapy, as blockade of EGFR is the first‐line therapy for patients with metastasized RAS/RAF‐wild‐type CRC, and combined blockade of EGFR and BRAF is first‐line therapy for patients with advanced CRC containing oncogenic BRAF mutations. We therefore asked whether targeted therapy is associated with changes in CRC cell states and trajectories. In addition to the RAS/RAF‐wild‐type organoid lines P009T and P013T, we employed OT227 and OT302 organoids carrying KRAS^G13D^ and KRAS^G12D^ mutations, respectively, and the BRAF^V600E^‐mutant and APC‐wt lines B2040 and C2019 (for panel sequencing, see Table EV2). The organoid lines exhibited different growth factor dependencies (Appendix Fig S6), but were uniformly cultured in the presence of EGF throughout the experiments for comparable results. As the blockade of EGFR using the antibody cetuximab was not effective *in vitro* as also observed by others (Schütte *et al*, 2017), we treated the organoids with the EGFR inhibitor Sapatinib, the BRAF inhibitor LGX818 (Encorafenib), the MEK inhibitor Selumetinib and combinations for 48 h, before subjecting single‐cell suspensions to CyTOF and scSLAM‐seq (Fig 5A, Appendix Fig S7 for summaries of CyTOF data) to measure relative activities of signal transducers and related transcriptional signatures, respectively (Fig 5B).

Inhibitor treatments had variable effects on the cell signaling network between lines, but we observed communalities between the lines with shared RAS/RAF mutational status (Fig 5C): In RAS/RAF‐wild‐type lines, treatment with EGFR inhibitor reduced both MEK and ERK phosphorylation. MEK inhibition decreased ERK phosphorylation, but increased MEK phosphorylation via negative feedback suppression (Fritsche‐Guenther *et al*, 2011). BRAF inhibitors raised levels of MEK and ERK phosphorylation, suggesting paradoxical activation of RAF (Hatzivassiliou *et al*, 2010). In contrast, the KRAS mutant lines OT227 and OT302 were largely unresponsive to EGFR inhibition, while MEK inhibition caused strong upregulation of MEK phosphorylation, and BRAF blockade strongly upregulated both MEK and ERK phosphorylation. A similar response to MEK inhibition was found in the BRAF‐mutant lines C0219 and B2040; however, in these lines BRAF inhibition alone or in combination with EGFR inhibition resulted in substantial loss of ERK phosphorylation.

Cell signaling changes translated into gene expression (Fig 5D), as we found high correlation between ERK phosphorylation and MAPK target gene activity (*P* < 0.0001, using linear models with line‐specific offsets; Fig 5E). Furthermore, across all CRC organoids, we observed a positive correlation between p‐ERK and KI67 protein levels (*P* < 0.005) and a negative correlation between p‐ERK and cleaved Caspase3 (*P* < 0.005) and cleaved PARP levels, in line with roles of MAPK in activation of proliferation and inhibition of apoptosis. Positive correlations also existed between p‐ERK and YAP (*P* < 0.0001), and between MAPK target expression and YAP target expression (*P* < 0.0001). In contrast, MAPK target activity was negatively correlated with Wnt target activity (and *P* < 0.05, respectively; Fig 5E). A direct interaction between MAPK and YAP signaling is supported by induction of transgenic BRAF^V600E^ in a mouse intestinal organoid model (Riemer *et al*, 2015), which resulted not only in activation of the MAPK target gene signature, but also in an even stronger activation of YAP target genes (Fig 5F).

### Targeted therapy alters developmental trajectories of CRC organoid cells

**Figure 6:**
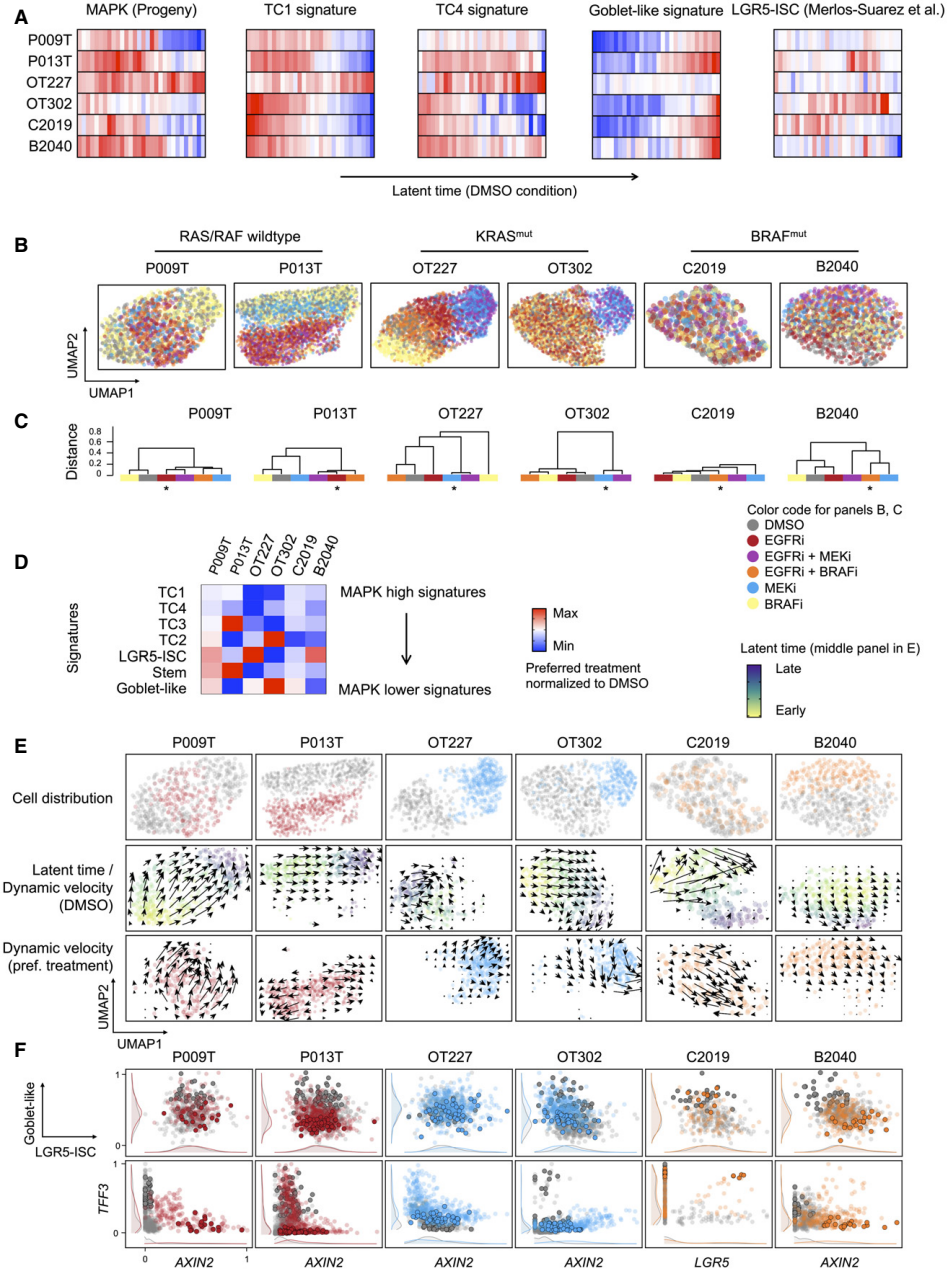
Anti‐MAPK therapy re‐routes developmental trajectories in CRC organoids Activities of gene expression signatures, as indicated, in single‐cell transcriptomes from control (DMSO) condition CRC organoids, ordered along latent time. Correlation between cell state distributions and latent time was calculated using Pearson’s r. For correlations and significances, see Table EV6. Color code red: high activity; blue: low activity.UMAPs of organoid single‐cell transcriptomes, color‐coded by treatment conditions, as indicated.Dendrograms of transcriptome similarities across treatment conditions, per organoid line. Height of dendrogram is obtained by hierarchical clustering on the overlap of KNN neighborhoods across conditions. Preferred treatment conditions are marked by asterisk.Heatmap of signature gene expression, from scRNA‐seq data. Average activities of gene signatures are given as log fold change for preferred treatment condition, after normalization to DMSO control condition. Range of color scale was adjusted for each signature.RNA velocity analysis of organoids under DMSO and preferred treatment conditions. Latent time is given as color code in the control (DMSO) condition UMAP, dynamical velocities are displayed as arrows.Scaled signature expression and single gene expression moments per cell, under control (gray) and preferred treatment (color) conditions, as indicated. 10% of cells populating the end of latent time are displayed in bold. It is of note that latent time is linear and thus cannot capture multiple populations located at developmental end points. Densities at the sidelines display expression in all cells.

We traced developmental trajectories in the panel of six CRC organoid lines by computing latent time from scSLAM data in the absence of treatment (DMSO control condition; Fig 6A). In five out of six organoids, cells at early latent time expressed significantly higher levels of MAPK targets and TC1 and TC4 signatures, extending our previous results to lines with mutations in the MAPK pathway. In contrast, cells at late latent time expressed the Goblet cell signature, indicating differentiation at trajectory endpoints (for statistics on cell state signature distributions, see Table EV6). The stem cell‐related LGR5‐ISC signature was not uniformly graded along latent time. The outlier line OT227 did not show clear directionality of cell development.

Anti‐MAPK therapies had broad consequences on gene expression, as quantified by hierarchical clustering of conditions based on a similarity matrix of shared neighboring cells (Fig 6B and C). We found that inhibition of EGFR, alone or in combination with BRAF or MEK inhibition, was most effective in the induction of transcriptome changes in the RAS/RAF‐wild‐type organoid lines P009T and P013T. A combination of BRAF and EGFR inhibitors deregulated transcriptomes effectively in the BRAF‐mutant B2040 and C2019 organoids, while both inhibitors had smaller effects on their own. As also observed for ERK phosphorylation (Fig 5C, above), these data agree with clinical reality, where positive outcomes for EGFR and combinatorial BRAF/EGFR inhibition are variable but limited to patients with RAS/RAF‐wild‐type and BRAF‐mutant CRC, respectively. Effective anti‐MAPK treatment contrasted with chemotherapy in outcomes regarding transcriptome regulation and cell cycle distribution in the organoids, indicating distinct mechanisms of action (Appendix Fig S8).

Based on the above results, we concentrated on anti‐EGFR, anti‐MEK, and combined anti‐EGFR/BRAF inhibition for RAS/RAF‐wild‐type, KRAS‐ and BRAF‐mutant organoids, respectively, and analyzed cell development and cell state prevalence for these preferred inhibitor combinations. Averaging across all cells, the MAPK‐high TC1 and TC4 cell state signatures were downregulated by the preferred treatments in all lines (Fig 6D). In P009T, P013T, OT227, and B2040 organoid lines, the MAPK inhibitors induced LGR5‐ISC or stem cell cluster‐related signatures, while OT302 showed stronger differentiation‐related Goblet cell signature gene expression (Fig 6F). To analyze whether gene expression changes have a basis in directional cell development, we first compared developmental trajectories, as visualized by dynamic velocities in the UMAPs. Trajectories were re‐routed to new endpoints in P013T, OT302, and C2019 organoids under treatment and were induced in the OT227 line that did not show directional development in the absence of treatment (Fig 6E). Cells at the ends of trajectories (marked in bold in Fig 6F) were distinguished from the bulk of cells by lower differentiation‐associated Goblet‐like gene expression in four out of six lines (P009T, P013T, OT302, and B2040), and the same cells showed higher stem cell‐ and Wnt‐associated LGR5‐ISC signature expression, or activation of Wnt targets or stem cell markers such as *AXIN2* or *LGR5* in some lines.

**Figure EV5:**
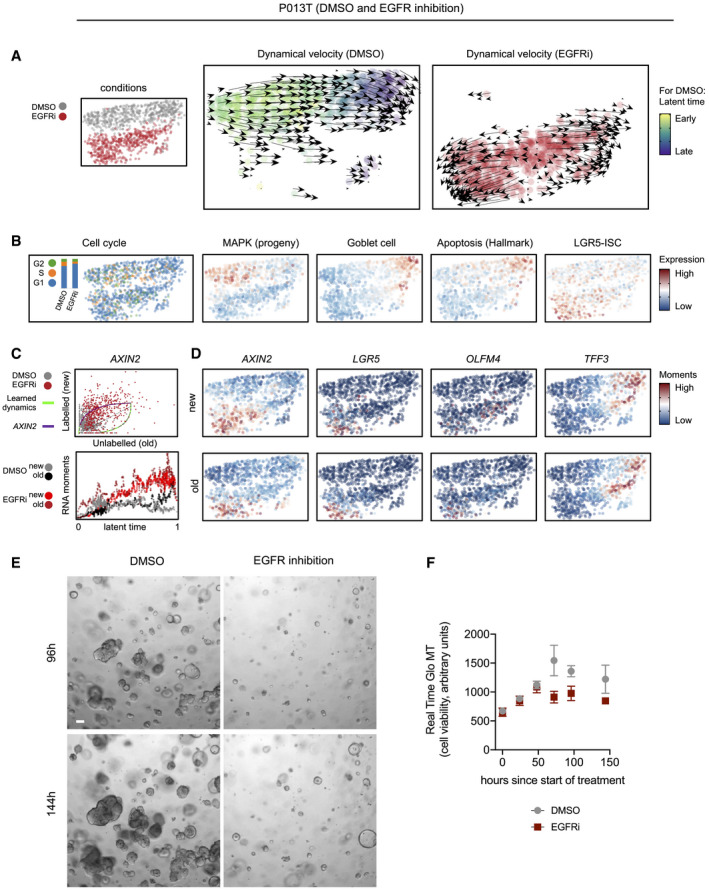
Detailed analysis of EGFR inhibition effects in P013T CRC organoids AUMAPs and dynamical velocity maps of the two conditions (DMSO versus EGFR inhibition), color‐coded by condition or latent time, as indicated. Latent time cannot be defined for EGFR inhibited cells showing divergent trajectories.BUMAPs of the two conditions, color‐coded by cell cycle phase or activities of selected gene expression signatures, as indicated.CPhase plot and latent time RNA moments for *AXIN2*.DUMAPs of the two conditions, color‐coded by gene‐specific expression moments.E, FOrganoid culture of P013T organoids reveals outgrowth of EGFR‐resistant cells. (E) Phenotypes of DMSO control and EGFR‐treated P013T cultures after 96 and 144 h, as indicated. Organoids growing under EGFR inhibition show a distinct spheroidal phenotype. Scale bar 100 µm (F) Metabolic cell viability of DMSO control and EGFR inhibitor‐treated P013T cultures. Graph displays mean and standard deviation of triplicate assays.

We analyzed cell state changes in detail in P013T organoids, where EGFR inhibition induced divergent developmental trajectories toward two distinct endpoints after 48 h of treatment (Figs 6E and EV5). A common endpoint between control and EGFRi conditions was characterized by goblet cell differentiation and the hallmark apoptosis signature, whereas a new treatment‐induced endpoint was characterized by LGR5‐ISC signature expression (Fig EV5A and B). Accordingly, trajectories toward the latter endpoint showed *de novo* expression of the Wnt targets *AXIN2*, *LGR5*, *OLFM4* as indicated by the dynamics in the phase plot after EGFR inhibition and also by the more restricted fields of expression for the older non‐labeled as compared to the newer labeled RNAs (Fig EV5C and D).

These gene activity patterns are strong indications for the re‐routing of cell development under MAPK inhibition, away from differentiation‐associated developmental endpoints toward a state high in expression of Wnt‐driven stem cell gene expression. The computational analyses agree with organoid culture phenotypes showing slow outgrowth of resistant P013T colonies under long‐term EGFR treatment (Fig EV5E and F).

## Discussion

Here, we provide a comprehensive analysis of patient‐overarching transcriptional states of CRC cells forming developmental trajectories. We find that cell hierarchies in CRC are organized along developmental trajectories following MAPK gradients. Our analyses imply that CRC cells develop gradually and directional rather than forming fixed cell populations in organoids and probably also in patients. As cell state prevalence differed between patients, we suggest that CRC cell trajectories are guided and constrained by individual cancer characteristics such as paracrine signals from the tumor microenvironment and patterns of oncogenic driver mutations. Our experiments in organoids indicate that therapies targeting the MAPK pathway reduce proliferation, but can also redirect developmental trajectories of CRC cells toward endpoints that are likely associated with therapy resistance.

Previous single‐cell studies defined features of the CRC immune microenvironment (James *et al*, 2020; Lee *et al*, 2020; Qian *et al*, 2020; Zhang *et al*, 2020). However, no consensus exists on patient‐overarching features defining cells of the epithelial CRC compartment. Here, we define six transcriptome‐based states of CRC cells, termed stem/TA‐like, goblet cell‐like, and TC1‐4, that have differential activities of oncogenic pathways. Individual stem cell markers such as *OLFM4* or *CD44* varied in expression between the stem/TA‐cell‐like and TC1‐4 clusters and were also found overexpressed in clusters of colorectal polyp and cancer cell of another study (preprint: Becker *et al*, 2021). Based on these transcriptional patterns, it appears that no unique stem cell signature exists in CRC and that Wnt, YAP, and MAPK activities together can maintain different cell states that may act as functional equivalents of stem cells that can sustain cancer growth. The TC1‐4 clusters were predominantly populated by copy number‐aberrant cancer cells but also contained some non‐cancerous epithelial cells. We therefore propose that, while oncogenic signals are main drivers of the TC1‐4 transcriptional states, (tumor‐)microenvironmental signals and cell‐intrinsic cues can result in normal epithelial cells to assume such transcriptional states, too.

Like many current single‐cell studies, our work is limited by rather small patient and organoid cohorts. Furthermore, our analyses focused on SCN‐aberrant CRC cells, and therefore, the heterogeneity of SCN‐stable CRC, in particular MSI tumors, is not covered. Community efforts integrating multiple studies will be required to provide a general framework of how genetic drivers and the microenvironment interact to direct cell state prevalence, developmental trajectories, and cell plasticity during tumor progression and under therapy. Such analyses may result in a consensus structure for single‐cell CRC transcriptomes different from the six CRC cell cluster model proposed here.

MAPK is a key pathway for targeted therapy, as many CRC patients profit from anti‐EGFR or anti‐EGFR/anti‐BRAF therapy (Amado *et al*, 2008; Karapetis *et al*, 2008; Kopetz *et al*, 2019). By and large, outcomes of our experimental inhibition of MAPK in organoids agreed with known relationships between predictive mutations and therapy sensitivity, as EGFR inhibition was only effective in RAS/RAF‐wild‐type organoids, while a combination of BRAF and EGFR inhibitors—but not each inhibitor alone—had profound effects on development of BRAF^V600E^‐mutant CRC organoids. In addition, we show that graded MAPK‐driven gene expression informs developmental trajectories, extending previous finding of graded ERK activity in CRC organoids (Brandt *et al*, 2019). Our analyses suggest that intrinsic resistance to anti‐MAPK therapies may rely on re‐routing of developmental trajectories of CRC cells. Indeed, the ability to reverse developmental trajectories in the intestinal epithelium has been found before (Buczacki *et al*, 2013; Schwitalla *et al*, 2013; Jadhav *et al*, 2017). Our study therefore adds new aspects to current models of anti‐MAPK therapy resistance defined by cell plasticity (Misale *et al*, 2014; Woolston *et al*, 2019; Lupo *et al*, 2020).

Our study suggests that multiple signaling pathways form a therapy‐relevant interconnected network of oncogenic signaling pathways in CRC. For instance, we find that YAP and MAPK levels are positively correlated on the protein activity and the transcriptional response levels. YAP maintains regenerative responses and is a key driver of CRC and other cancers (Zanconato *et al*, 2016). In contrast, MAPK and Wnt signaling were negatively correlated. We and others previously showed loss of Wnt‐driven intestinal stem cells by high MAPK levels provided by oncogenic BRAF (Riemer *et al*, 2015; Tong *et al*, 2017). Here, we find that therapeutic inactivation of MAPK can result in Wnt and LGR5‐ISC signature reactivation in CRC, confirming two previous studies (Zhan *et al*, 2019; Lupo *et al*, 2020). As both pathways, Wnt and MAPK, are generally activated by oncogenic mutations in CRC, cross‐inhibition between the pathways would mean that therapeutical suppression of one pathway results in oncogene‐driven activation of the other, possibly explaining why many therapeutic approaches to block MAPK proved insufficient in the clinic. It will be an important goal for future studies to identify combinations of actionable signals that can be exploited for therapies resulting in uniform commitment of CRC cells to differentiation‐related and apoptotic endpoints instead of re‐routing subsets of cancer cells toward stem cell‐like states.

Our study also identified further cancer traits in CRC cell clusters with relevance to therapy. For instance, TC1 cells were defined by high levels of replication stress, which can be functionally associated with high MAPK activity (Sheu *et al*, 2012; Klotz‐Noack *et al*, 2020). Tumors with high TC1 cell content were strongly positive for PARP, an important therapeutic target (Sun *et al*, 2020). It is of note that the CMS subtyping system developed for bulk tissue CRC transcriptomes (Guinney *et al*, 2015) could not distinguish the CRC cell types that we identified here on the single‐cell level, as most epithelial cancer cells were assigned to CMS1 or CMS2 with the exception of goblet‐like CRC cells that can adopt CMS3 (Appendix Fig S9).

In addition to patient‐overarching CRC cell traits, we also observed patient‐specific gene expression differences. Our integrated analysis of single‐cell transcriptomes and copy number gains and losses indicated that patient‐specific gene expression patterns were significantly associated with copy number gains that we inferred from transcriptomes and validated using exome sequencing of three patients. On another level, we observed patient‐specific gene expression patterns that translated into regional patterns of proteins, as evidenced by the staining for MMP7 which was confined to the invasive front of some tumors. Such patterns of gene activity and protein translation indicate that tumor cell development is highly plastic and partly regulated by immune and stromal cells in the microenvironment. Thus, we expect that the intrinsic developmental paths of CRC cells that we observe in organoid cultures are modulated by extrinsic cues from the microenvironment *in vivo*. Future studies using co‐cultures of different tumor‐associated cell types could disentangle key paracrine relationships in cancer.

We analyzed here primary cancer epithelium and organoid transcriptomes. Novel single‐cell approaches, taking into account the diversity of the tumor microenvironment in patient cohorts stratified by treatment, complex cell culture models, the extension of single‐cell analyses to multi‐omics, and the preservation of spatial information at a cellular level, promise to identify cellular heterogeneity and genetic diversity of cancer at even greater detail in the future. The combination of such approaches has the potential to improve the molecular understanding of cancer and therapy prediction for patients (Rajewsky *et al*, 2020). Our work defining developmental trajectories of CRC contributes to this goal.

## Materials and Methods

### Collection and single‐cell RNA sequencing of clinical specimens

Fresh normal colon and colorectal cancer tissues were acquired during the intraoperative pathologist's examination at Charité University Hospital Berlin. Tissues (approx. 0.1–0.4 g) were minced using scalpels, processed using the Miltenyi Human Tumor Dissociation Kit (Miltenyi, #130‐095‐929) and a Miltenyi gentleMACS Tissue Dissociator (Miltenyi, #130‐096‐427), using program 37C_h_TDK_1 for 30–45 min. For three tumors, we also used digestion with the cold active protease from Bacillus licheniformis (Sigma P5380) at approx. 6°C for 45 min. with frequent agitation, following a published protocol (Adam *et al*, 2017) (Appendix Fig S10). Cell suspensions were filtered using 100 µm filters, pelleted by centrifugation, treated with 1 ml ACK erythrocyte lysis buffer, washed and resuspended in ice‐cold PBS, and filtered using 20 µm filters. Debris was removed using the Debris Removal Solution (Miltenyi #130‐109‐398). Cell suspensions were analyzed for cell viability > 75% using LIVE/DEAD Fixable Dead Cell Stain Kit (488 nm; Thermo Fisher) and a BD Accuri cytometer. 10,000 single cells were used for single‐cell library production, using the Chromium Single‐Cell 3´Reagent Kits v3 and the Chromium Controller (10× Genomics). Libraries were sequenced on a HiSeq 4000 Sequencer (Illumina) at 200–400 mio. reads per library to a mean library saturation of approx. 50%. This resulted in 35,000 to 120,000 reads per cell.

### DNA sequencing

For panel sequencing, DNA was extracted from FFPE tumor tissue using the Maxwell RSC DNA FFPE Kit (Promega) or the GeneRead DNA FFPE kit (Qiagen) and sequenced using a CRC panel (Mamlouk *et al*, 2017), and/or the Ion AmpliSeq Cancer Hotspot Panel (CHP) v2 and an IonTorrent sequencer (Thermo Fisher). Variant calling was performed using Sequence Pilot (Version 4.4.0, JSI Medical Systems) or SoFIA (Mamlouk *et al*, 2017). For exome sequencing, DNA was isolated from fresh‐frozen tumor tissue using the DNeasy Blood and Tissue Kit (Qiagen). Exomes were sequenced using the AllExon Human SureSelect v7 Kit (Agilent).

### Histology and immunostaining

3–5 µm tissue sections of formalin‐fixed and paraffin‐embedded (FFPE) tissue were used. Immunostainings were performed on the BenchMark XT immunostainer (Ventana Medical Systems), using CC1 mild buffer or Ultra CC1 buffer (Ventana Medical Systems) for 30 min at 100°C, and using antibodies rabbit anti‐TFF3 (1:250, Abcam, ab108599), mouse anti‐FABP1 (1:1,000, Abcam, ab7366), rabbit anti‐OLFM4 (1:100, Atlas Antibodies, HPA077718), mouse anti‐EPCAM (1:100, Thermo Scientific, MS‐144‐P1), rabbit anti‐Ki67 (1:400, Abcam, ab16667), mouse anti‐Ki67 (1:50, Dako, M7240), rabbit anti‐LYZ (1:1500, Abcam, ab108508), rabbit anti‐EREG (1:50, Thermo Fischer Scientific, PA5‐24727), anti‐PARP1, mouse anti‐MUC2 (1:50, Leica, NCL‐MUC‐2), mouse anti‐CK17 (1:10, Dako, M7046), and mouse anti‐MMP7 (1:100, Thermo Fisher Scientific, MA5‐14215). Images were taken using AxioVert.A1 (Zeiss) or CQ1 (Yokogawa) microscopes or scanned using the Pannoramic SCAN 150 scanner (3DHISTECH).

### Organoid culture and metabolic labeling

Tumor cells were washed in Advanced DMEM/F12 medium (Gibco), embedded in Matrigel, and cultured in 24‐well plates, as published (Sato *et al*, 2011). Wnt3 and R‐Spondin3 were prepared as conditioned media (Sato *et al*, 2011). For testing of media conditions, organoids were re‐plated four days after disaggregation into a 96‐well plate and grown in the different media (no growth factors; +EGF; +Wnt/R‐Spondin/Noggin/EGF; +AZD8931, 100 nM). After 6 days, cell viability was measured with Real‐Time‐Glo MT Cell Viability Assay (Promega, G9712) and Cyto3D Live‐Dead Assay Kit (The Well Biocioscience, BM01). NCO, P009T, and P013T replicate cultures were cultured in media with and without Wnt/R‐Spondin, and P009T, P013T, OT227, OT302, B2040, C2019 organoids were cultured in standard media (Sato *et al*, 2011; Schütte *et al*, 2017) with DMSO or were treated for 48 with 100 nM AZD8931 (Sapatinib), 100 nM LGX818 (Encorafenib) and/or 8 µM AZD6244 (Selumetinib), 5 µM 5‐fluoro‐uracil or 5 µM oxaliplatin. For single‐cell sequencing, organoids were dissociated completely using TrypLE and DNAseI, and filtered via a 20 µm filter. For single‐cell SLAM‐seq, organoids were metabolically labeled in culture using 200 µM 4‐thio‐uridine for 2 h (Herzog *et al*, 2017), harvested, disaggregated to single cells by TrypLE, and fixed in fixation buffer (80% methanol/20% DPBS) at ≥ −20°C. Samples were warmed to room temperature and incubated with 10 mM iodoacetamide. Alkylation was carried out overnight, in the dark, with gentle rotation, followed by two washes with cold fixation buffer. Single‐cell suspensions were rehydrated and incubated 10 min at room temperature in 100 mM DTT. Samples were resuspended in fixation buffer and conserved at −80°C.

### Mass cytometry (CyTOF)

For CyTOF analysis, we used a panel of antibodies described in Brandt *et al*, 2019, and measurements were performed essentially as in described in the same publication. In short, organoids were harvested in PBS and digested to a single‐cell solution in 1:1 Accutase (BioLegend) and TrypLE (Gibco) with addition of 100 U/ml Universal Nuclease (Thermo Scientific) at 37°C. Cells were counted, and a maximum of 500,000 cells were stained with 5 µM Cell‐ID Cisplatin (Fluidigm) in PBS for 5 min at 37°C, washed in PBS, resuspended in medium and incubated for 30 min at 37°C, resuspended in BSA/PBS solution, mixed 1:1.4 with Proteomics Stabilizer (Smart Tube Inc.), and frozen at −80°C for storage.

For analysis, cells were thawed, mixed with Maxpar Cell Staining Buffer (CSB, Fluidigm), labeled using the Cell‐ID 20‐Plex Pd Barcoding Kit, and washed again in CSB, then in Barcode Perm Buffer (Fluidigm). After barcoding, cells were pooled and stained with a surface antibody cocktail, as described previously (Brandt *et al*, 2019). Data were acquired on a Helios CyTOF system. Mass cytometry data were normalized using the Helios software, and bead‐related events were removed. Doublets were excluded by filtering for DNA content (^191^Ir and ^193^Ir) vs. event length, and apoptotic debris removed by a filter in the platin channel (^195^Pt). De‐convolution of the barcoded sample was performed using the CATALYST R package version 1.5.3 (Chevrier *et al*, 2018).

### Primary tissue single‐cell RNA‐seq data analysis

UMIs were quantified using cellranger 3.0.2 with reference transcriptome GRCh38. Cell cycle was scored based on a list of cell cycle‐associated genes (Kowalczyk *et al*, 2015), and differences between S and G2 M expression scores were linearly regressed out per gene. For parameters and initial quality controls, see Appendix Fig S2. Epithelium, stromal, and immune cells were identified by scoring cell type markers across Louvain clusters for each sample (resolution = 1). Cell type markers used to score epithelium, stromal, and immune cells were adapted from Smillie *et al* (2019) and are listed in Table EV3. Sample‐wise quality control assessments and subsettings into main cell types are documented at sys‐bio.net/sccrc/. Normalized subsets were merged for each main cell type of normal and tumor samples without further batch correction.

SNN graph, Louvain clusters, and UMAP embeddings were recomputed for each subset based on top ten components. Louvain cluster‐specific marker genes of merged normal and tumor samples were used to identify sub‐cell types among epithelial, stromal, and immune subsets. Here, marker genes were determined with Seurat (Stuart *et al*, 2019) at a minimum log fold change threshold of 0.25. Gene expression sets were taken from the hallmark signature collection of the Broad institute (Liberzon *et al*, 2015), unless otherwise referenced in the main text, and were scored as implemented in the progeny R package and Seurat v3, respectively.

For copy number assessment, InferCNV v1.3.3 was used with default parameters. Copy number‐aberrant clones were cut at *k* = 2 in inferCNV dendrograms. Clone‐wise SCNA scores were computed by calculating the average standard deviation in inferCNV expression of all cells and divided by the average standard deviation in inferCNV expression of all normal samples taken together. Clones with a SCNA score greater than the highest observed score for normal samples were considered copy number‐aberrant.

For analysis of scRNA‐seq validation datasets, we downloaded publicly available data from the European Genome‐phenome Archive database (EGAS00001003779, EGAS00001003769) and ArrayExpress (E‐MTAB‐8410, E‐MTAB‐8412; Lee *et al*, 2020; Qian *et al*, 2020). We preprocessed data using the same pipeline as for our primary tissue analysis, including copy number calling and pathway activity scoring for the set of Belgian patients (KUL01‐KUL31). Additionally, we used the ingest function from scanpy to perform linear domain adaptation for asymmetric data integration. In short, ingest utilizes the PCA previously fitted onto our data to transform the reference dataset into the same space. Within this space, ingest can integrate the reference data into our UMAP coordinate system (Fig EV3C) and assign reference cells into corresponding cluster identities in the original dataset based on KNN classifiers (Fig EV3D–F).

### Organoid single‐cell RNA‐seq data analysis

Single‐cell SLAM sequencing data were preprocessed using cellranger v4.0, and labeled and unlabeled reads were counted using the alignments (as BAM files), using a custom pipeline utilizing Snakemake (Köster & Rahmann, 2012), SeqAn (Döring *et al*, 2008), R. For each read, the numbers of T nucleotides and T‐to‐C conversions were counted, leaving out positions with common SNPs (using the dbSNP build 151 as available as track from UCSC genome browser). For each molecule as identified by cell barcode and UMI, positions with discordant nucleotides were excluded. Subsequently, molecules were counted as nascent RNA if they contained a T‐to‐C conversion and old RNA otherwise.

scRNA‐seq and scSLAM‐seq data for organoids were analyzed using scanpy (Wolf *et al*, 2018)and scvelo (Bergen *et al*, 2020). For diffusion map analysis and RNA velocity, cells were first filtered by the number of genes (between 2,000 and 5,000) and the percent mitochondrial reads (between 0.075 and 0.2) and normalized, using scvelo standard settings. Cell cycle was scored and regressed out as for the primary cell data. UMAP embeddings were computed based on a PCA on only the 2,000 most highly variable genes obtained with scanpy. The similarity measure between conditions in Fig 6B was defined by the average fraction of shared neighbors in a nearest neighbor graph over all cells in the dataset, then performing hierarchical clustering on the resulting similarity matrix.

Moments were calculated on 30 principal components and 30 neighbors, separately per condition to avoid smoothing effects between different conditions within the same dataset. Velocity was calculated using the dynamical model from scvelo on 2,000 most highly variable genes according to scanpy, which were then filtered for at least 20 shared counts in both SLAM layers. Per gene, we usually observed either induced or repressed expression dynamics only. To resolve ambiguity in fitting kinetics to one‐sided dynamics, we modified the dynamical model from scvelo with an additional regularization term for the experiments analyzing Wnt‐ versus MAPK‐driven velocity, penalizing the number of cells that are assigned a higher latent time than a given root prior.

### Ethics permission

All patients were aware of the planned research and agreed to the use of tissue. Research was approved by vote EA4/164/19 of the ethics commission of Charité—Universitätsmedizin Berlin. Experiments conformed to the WMA Declaration of Helsinki and to the principles set out in the Department of Health and Human Services Belmont Report.

### Statistical analysis

Statistical analyses of single‐cell data were performed using non‐parametric Wilcox and Kruskal–Wallis tests, as indicated in main text and figure legends. Cell state distribution statistics (Fig 4C) were calculated using Pearson’s correlation coefficient (Pearson´s r), and significance of correlations was assessed by t statistics (Table EV6). Prevalence of deregulated genes in CNA genomic regions was calculated using Bonferroni‐corrected hypergeometric distribution and human genome GRCh38 gene numbers per chromosome arm (Fig S4). PARP immunofluorescence (Fig 2D) and organoid growth assays (Fig S6) were analyzed by unpaired t‐tests. Bulk tissue gene expression after transgene induction was analyzed by ratio paired t‐test (Fig 5F).

## Author contributions

PB, FU, SP, MM, AS, MLu, AT, TS, RA, YR, SM, KK‐N conducted and analyzed experiments; FU, SP, NB, BO, EB, T‐TW performed bioinformatic analyses; MM, NB, PB, CS, DB, BS, DH, MLa, RFS conceived, designed, interpreted experiments, and/or supervised parts of the study; PB, MM, DH, CK, AT contributed to clinical sample acquisition and preparation; MM wrote the manuscript; all authors provided critical feedback and helped shaping the research, analysis, and manuscript.

## Conflict of interest

The authors declare that they have no conflict of interest.

## Supporting information



Appendix

Expanded View Figures PDF

Table EV1

Table EV2

Table EV3

Table EV4

Table EV5

Table EV6

## Data Availability

Scripts for processing of patient tissue scRNA sequencing data are available from https://github.com/molsysbio/sccrc. Scripts for processing of organoid RNA velocity data are available from https://github.com/molsysbio/sccrc_slamvelocity. The datasets produced in this study are available in the following databases: patient sequencing data GEO GSE166555 (https://www.ncbi.nlm.nih.gov/geo/query/acc.cgi?acc=GSE166555), organoid sequencing data GEO GSE166556 (https://www.ncbi.nlm.nih.gov/geo/query/acc.cgi?acc=GSE166556). Processed count data are available from https://sys‐bio.net/sccrc.
