# Supplementary material for: Mitogen‐activated protein kinase activity drives cell trajectories in colorectal cancer
Source: EMBO Mol Med. 2021 Aug 19;13(10):e14123. doi: 10.15252/emmm.202114123 (PMC8495451; doi:10.15252/emmm.202114123)
Supplement: Supplementary file 2 — Expanded View Figures PDF [file EMMM-13-e14123-s008.pdf]

Expanded View Figures

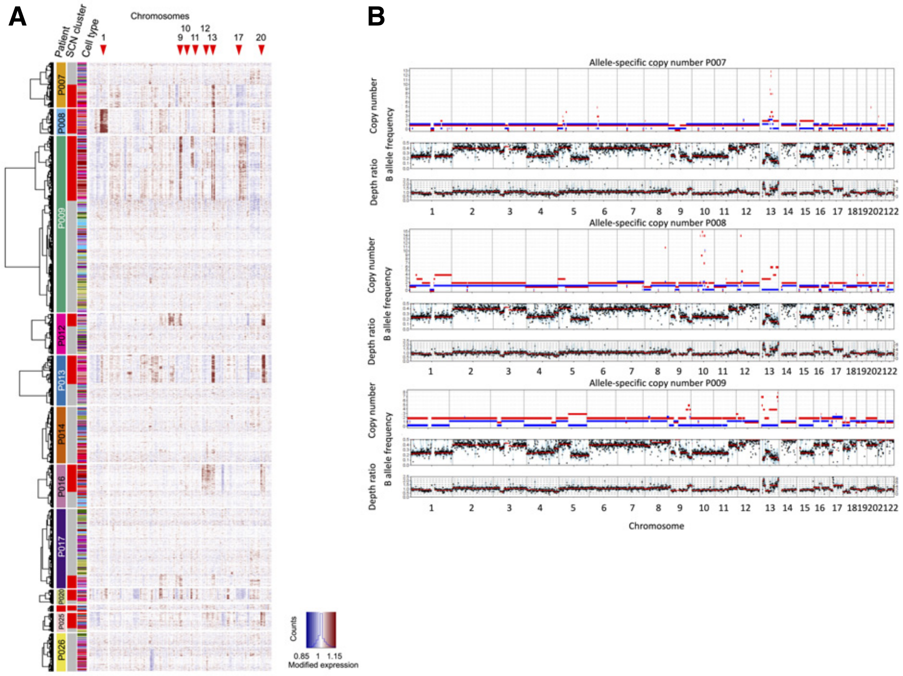

Figure EV1. Assessment of copy number variations.

A Copy number calling from single-cell transcriptomes, using InferCNV.

B Copy number calling from exome sequences. To validate SCNAs from single-cell transcriptome data, we performed allele-specific SCNA calling for patients P007, P008, and P009 from bulk whole exome data. Germline variants were discovered *de novo*, and read counts were accumulated for each allele at heterozygous germline variants using bcftools v1.9 multi-allelic caller. Discovered variants and read counts were passed to Sequenza 3.0 for segmentation and calling of allele-specific SCNAs. Top tracks: copy number segments of major and minor alleles. Middle tracks: B (minor) allele frequency. Bottom tracks: depth ratio of matched tumor versus normal samples with corresponding second axis of inferred copy number. P007 showed multiple LOH events on chromosomes 1, 3, 5, 6, 8, 9, 13, 15, 17, and 21 as well as a focal amplification on chromosome 13. P008 demonstrated a similar SCNA pattern with LOH events on chromosomes 8, 9, 10, 12, 13, 17, and 22 as well as multiple focal amplifications on chromosomes 8, 10, 12, and 13. P009 was characterized by widespread copy number neutral LOH in about 50% of the genome. Focal amplifications were found on chromosomes 9 and 13.

 © 2021 The Authors

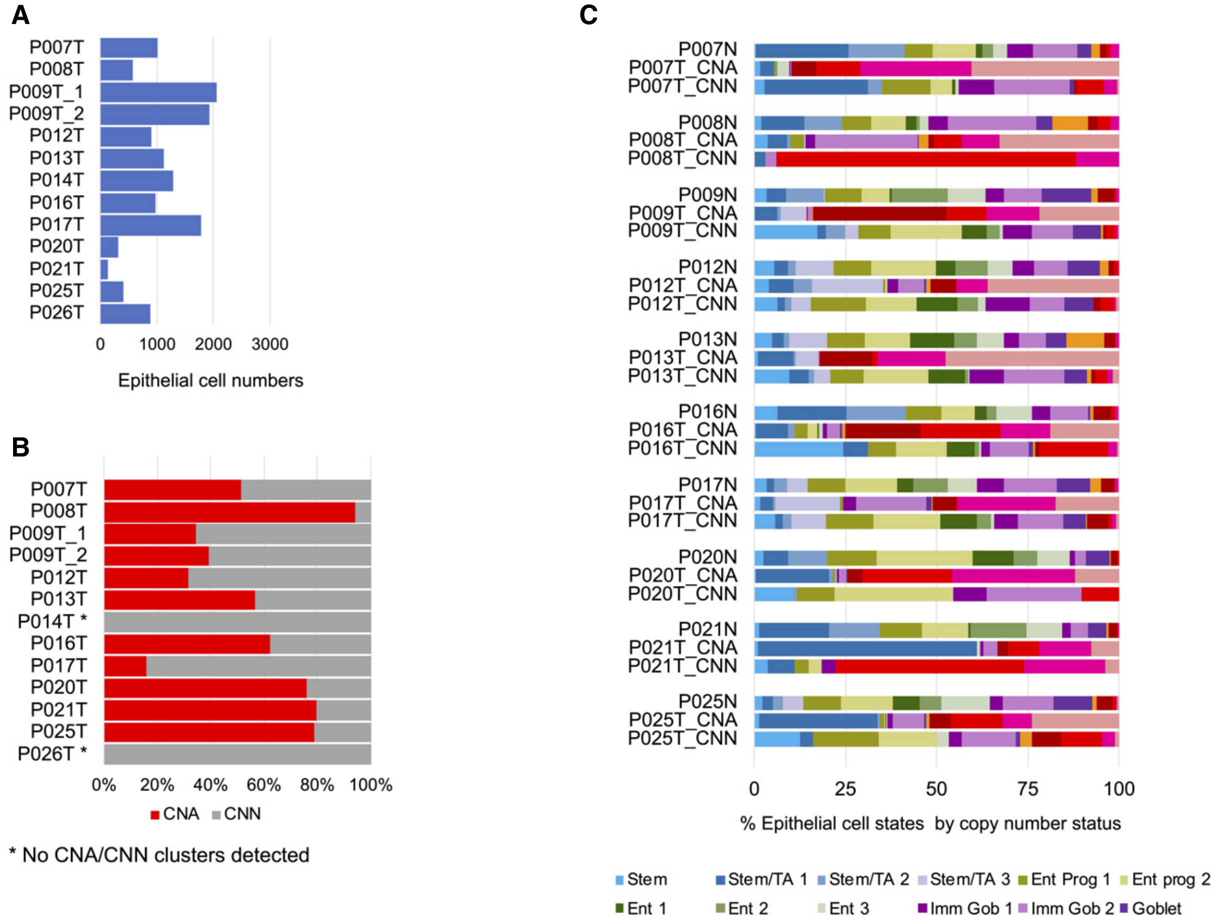

Figure EV2. Cell type census, per patient.

A Epithelial cell numbers per tumor sample retrieved by our analysis pipeline.

B Copy number calling on tumor samples. No copy number clusters could be called for P014T and P026T (see also Fig EV1A).

C Relative prevalence of cell differentiation states in the epithelial compartment, per patient. Cell state prevalence from tumor samples is given for copy number-aberrant (CNA) versus copy number normal (CNN) cells.

Figure EV3. Analysis of scRNA-seq data from validation cohorts.

A–F Analysis of the KUL01-KUL31 cohort (Lee *et al*, 2020; Qian *et al*, 2020). (A) Epithelial cell numbers per sample retrieved by our analysis pipeline. (B) Copy number calling on tumor border (TB) and tumor center (TC) samples. (C) Distribution of KUL01-KUM31 transcriptomes on the reference UMAP. Top left: blue: KUL01-KUL31 transcriptomes. Orange: Transcriptomes from P007-P026 samples; top right: Distribution of KUL01-KUM31 transcriptomes, by patient; bottom left: distribution of copy number normal versus aberrant transcriptomes; bottom right: distribution of tumor border versus tumor center transcriptomes. (D) Signature strengths of transcriptome clusters from our study (P007-P026), compared to KUL01-KUL31 transcriptome clusters after imputation. (E) Cell state distribution in the KUL01-KUL31 cohort, by category. (F) Cell state distribution in the KUL01-KUL31 cohort, by sample.

G Cell state distribution in the SMC01-SMC25 cohort (Lee *et al*, 2020), by sample.

© 2021 The Authors

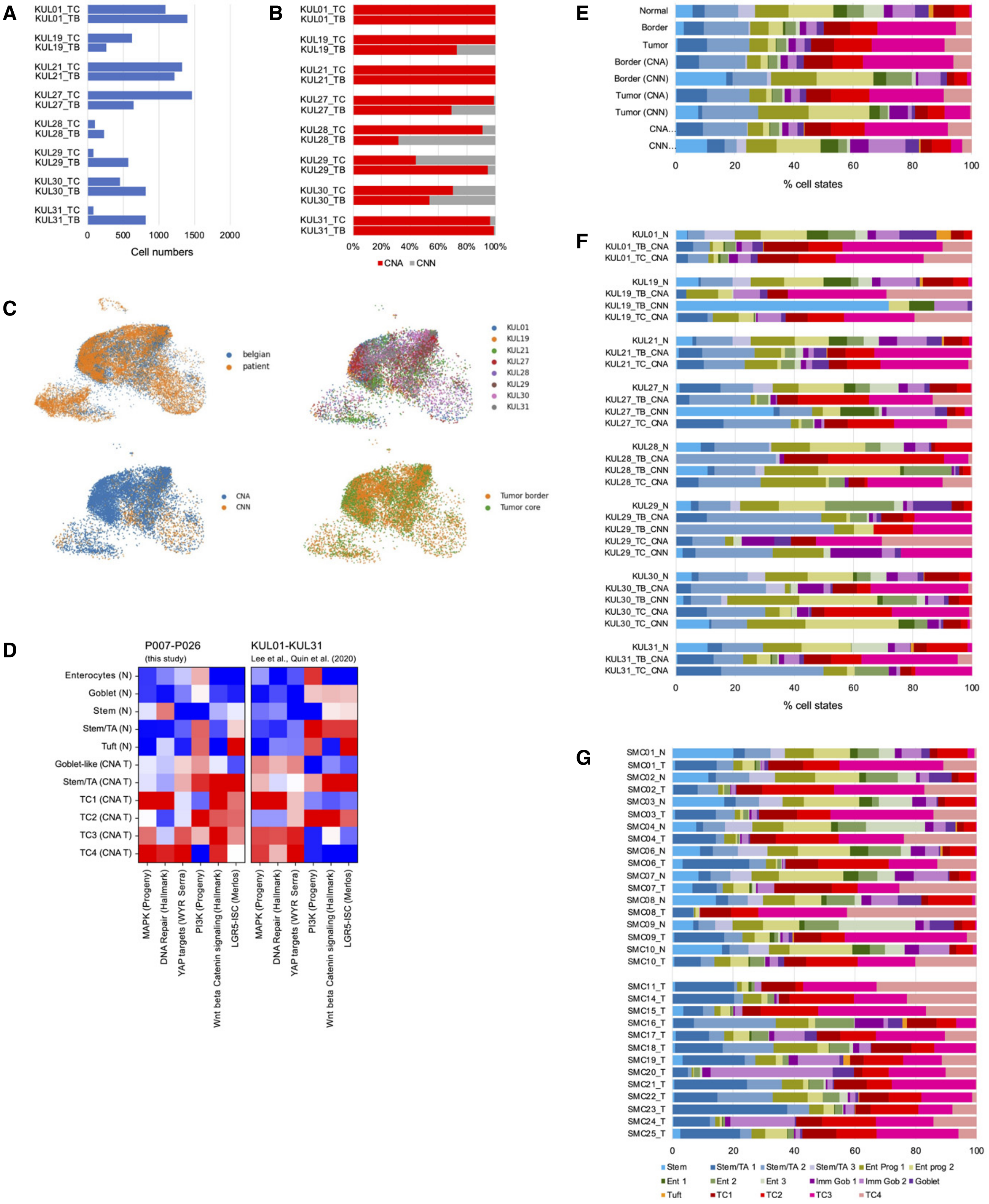

Figure EV3.

© 2021 The Authors

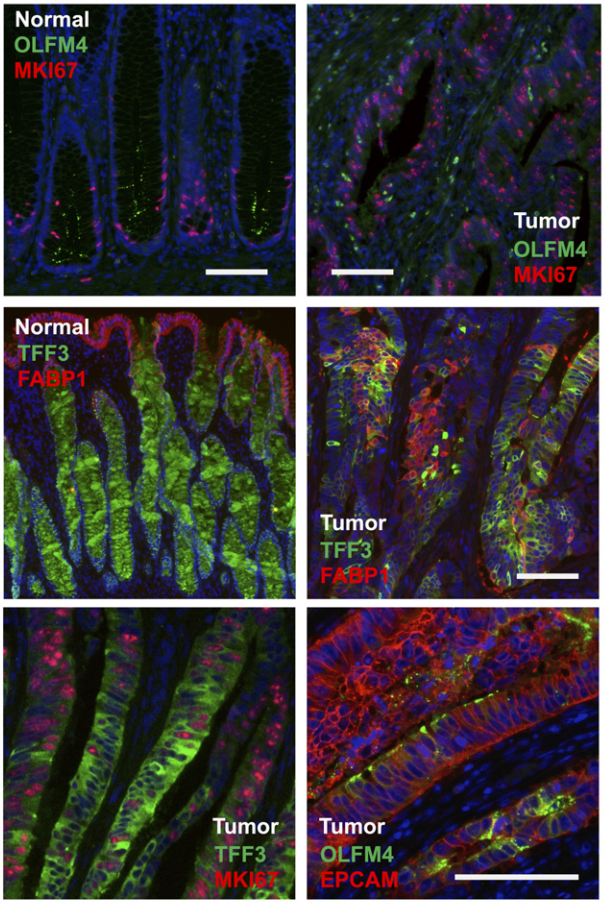

Figure EV4. Assessment of CRC cell heterogeneity by immunofluorescence.

Immunofluorescence analysis for OLFM4, MKI67, FABP1, and TFF3 in normal and tumor tissue. All sections are from patient P009, except the EPCAM/OLFM4 co-staining that was done on tumor tissue of P016. Scale bars indicate 100 μm. For marker expression in UMAP space, see Appendix Fig S3.

Figure EV5. Detailed analysis of EGFR inhibition effects in P013T CRC organoids.

- A UMAPs and dynamical velocity maps of the two conditions (DMSO versus EGFR inhibition), color-coded by condition or latent time, as indicated. Latent time cannot be defined for EGFR inhibited cells showing divergent trajectories.
- B UMAPs of the two conditions, color-coded by cell cycle phase or activities of selected gene expression signatures, as indicated.
- C Phase plot and latent time RNA moments for *AXIN2*.
- D UMAPs of the two conditions, color-coded by gene-specific expression moments.
- E, F Organoid culture of P013T organoids reveals outgrowth of EGFR-resistant cells. (E) Phenotypes of DMSO control and EGFR-treated P013T cultures after 96 and 144 h, as indicated. Organoids growing under EGFR inhibition show a distinct spheroidal phenotype. Scale bar 100 μm (F) Metabolic cell viability of DMSO control and EGFR inhibitor-treated P013T cultures. Graph displays mean and standard deviation of triplicate assays.

© 2021 The Authors

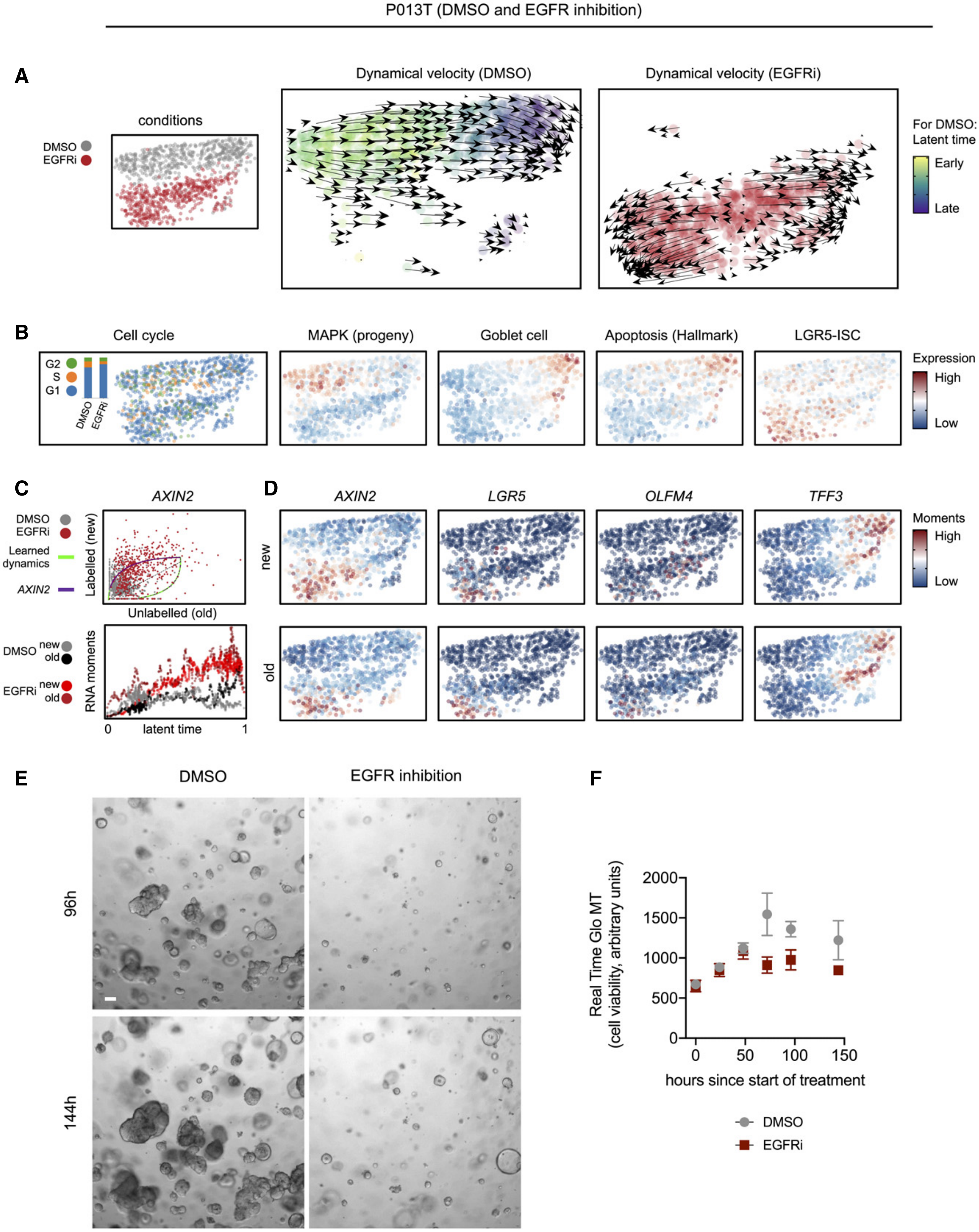

Figure EV5.

 © 2021 The Authors
