# Supplementary material for: Mitogen‐activated protein kinase activity drives cell trajectories in colorectal cancer
Source: EMBO Mol Med. 2021 Aug 19;13(10):e14123. doi: 10.15252/emmm.202114123 (PMC8495451; doi:10.15252/emmm.202114123)
Supplement: Supplementary file 9 [file EMMM-13-e14123-s002.pdf]

EMBO Molecular Medicine

Mitogen-activated protein kinase activity drives cell trajectories in colorectal cancer

Florian Uhlitz, Philip Bischoff, Stefan Peidli, Anja Sieber, Alexandra Trinks, Mareen Lüthen, Benedikt Obermayer, Eric Blanc, Yana Ruchiy, Thomas Sell, Soulafa Mamlouk, Roberto Arsie, Tsu-Ting Wei, Kathleen Klotz-Noack, Roland Schwarz, Birgit Sawitzki, Carsten Kamphues, Dieter Beule, Markus Landthaler, Christine Sers, David Horst, Nils Blüthgen, and Markus Morkel
DOI: 10.15252/emmm.202114123

Corresponding authors: Markus Morkel (markus.morkel@charite.de) , Nils Blüthgen (nils.bluethgen@charite.de)

Review Timeline:

Submission Date:	11th Feb 21
Editorial Decision:	9th Mar 21
Revision Received:	1st Jun 21
Editorial Decision:	20th Jul 21
Revision Received:	27th Jul 21
Accepted:	30th Jul 21

Editor: Zeljko Durdevic

Transaction Report:

(Note: With the exception of the correction of typographical or spelling errors that could be a source of ambiguity, letters and reports are not edited. Depending on transfer agreements, referee reports obtained elsewhere may or may not be included in this compilation. Referee reports are anonymous unless the Referee chooses to sign their reports.)

9th Mar 2021

Dear Dr. Morkel,

Thank you for the submission of your manuscript to EMBO Molecular Medicine. We have now received feedback from the two reviewers who agreed to evaluate your manuscript. As you will see from the reports below, the referees acknowledge the interest of the study but also raise serious concerns that should be addressed in a major revision.

Addressing the reviewers' concerns in full will be necessary for further considering the manuscript in our journal, and acceptance of the manuscript will entail a second round of review. EMBO Molecular Medicine encourages a single round of revision only and therefore, acceptance or rejection of the manuscript will depend on the completeness of your responses included in the next, final version of the manuscript. For this reason, and to save you from any frustrations in the end, I would strongly advise against returning an incomplete revision.

We realize that the current situation is exceptional on the account of the COVID-19/SARS-CoV-2 pandemic. Therefore, please let us know if you need more than three months to revise the manuscript.

I look forward to receiving your revised manuscript.

Yours sincerely,

Zeljko Durdevic
Editor
EMBO Molecular Medicine

***** Reviewer's comments *****

Referee #1 (Comments on Novelty/Model System for Author):

confusion between cell types within one organoid going into different differentiation states, versus these effects across patients.
diversity within organoid can be model artefact and patient signal cannot be determined in small study

Referee #1 (Remarks for Author):

In line with initial reviewer 2, I still have concerns about the strong generalized statements on patient independent common epithelial cell types . Resolution, sample size, patient cohort, all may be impacting some of the initial findings that serve as a backbone many further statements
The authors strongly state :
(i) cells from all twelve tumors form common clusters, in particular the TC1-4 clusters, suggesting strong commonalities between CRCs of different origins or progression histories.
(ii) (ii) Absorptive differentiation appears to be universally blocked despite different progression histories.
(iii) (iii) copy number changes appear to responsible for individual gene expression differences, see below.
(iv) (iv) CRC tumor cell types appear to cluster by MAPK strength in all tumors
And: "In summary, our analyses define normal stem/TA-like cells, immature goblet-like cells, and TC1-4 cells as six main patient-overarching clusters of CRC cells.
This overall 6 group structure is important as the next set of experiments ranks the groups in terms of MAPK activation and through interventions suggest that these different states are MAPK driven
This is an interesting concept, but to fit more with the current literature and guide the reader, it would be good to have more perspective on
- The 'validity' of the 6 groups. Please compare to the many other CRC single cell studies and their conclusion on number and type of patient independent signal
- The more is known about the T1-4groups, the more we can proceed in a focused way to understand them . Comparison to similarly generated subgroups described in other papers would be useful.
- There are also premalignant polyp single cell data with patient independent groups and overlapping markers to those used here, would be good to add to the discussion
- The functional work in organoids here with MAPK inhibition and velocity to study causality has intrinsic value in establishing the concept of MAPK drive, but the more the paper can put the groups of cell types in perspective and increase confidence in their precise definitions, the more valuable the MAPK work becomes.
Line 83 add the relevant plasticity under therapy work of Lupo et al., Sci. Transl. Med. 12, eaax8313 (2020) 5 August 2020
Line 286 and line 318 onwards ". Our findings thus indicate that the MAPK, YAP and Wnt signaling pathways form an interconnected network controlling transcription, and ultimately proliferation and cell fate in CRC" please refer to the same paper, that already details this concept and incorporate their data in perspective here.
Discussion line 358 ' that therapies targeting the MAPK pathway 359 reduce proliferation, but also redirect developmental trajectories of CRC cells towards goblet-cell 360 differentiation-like apoptotic or Wnt-driven stem-cell like endpoints that could be associated with 361 therapy-sensitivity or resistance, respectively. In summary, our analysis provides a single cell-based 362 framework for cell plasticity during cancer development and targeted therapy.' These broad statements are not very useful, certainly in light of
1. The very detailed work by Lupo mentioned above (which therapy in which patient will give what effect)
2. The small cohort here that cannot reliable detect patterns
3. The wild type versus mutation status not taken into account into such statements

Referee #2 (Comments on Novelty/Model System for Author):

This manuscript uses single cell RNAseq of 12 tumor samples and some functional experiments with organoids (6 samples) to better characterize colorectal cancers. Their single cell RNAseq analysis of tumor material identified 6-7 cell phenotypes to be present in mutated (as measured by CNA) epithelial cells. This includes stem and TA cells, immature secretory (goblet) cells and four populations which are more tumor specific: TC1=cells in G2/S and with increased replication stress, TC2 = cells with high PI3K activity, T3=cells without mentioned signature, and TC4 = cells with high YAP target signature. Both, TC1 and TC4 show also high ERK/MAPK target signatures. The authors then continue to perform functional experiments with 2 organoid samples derived from these primary tumors and 4 other organoid samples described previously. They conclude that MAPK signalling affects the differentiation state in these tumors and that EGFR/MEK/BRAF inhibition can affect this.

Overall, the manuscript is largely descriptive, the functional data on EGFR/BRAF/RAS inhibitors is not convincing and has either to be expanded with more organoids or their claims have to be toned down.
Basically all of their observations have already been published. Their observation that MAPK inhibition can affect intestinal tumorigenesis is not novel and MEK as well as Erk inhibitors have already previously been shown to reduce tumor formation in APC mutants (https://doi.org/10.1111/cas.12670; DOI: 10.1038/nm.2143).
But as Michael Karin has formulated it already in his paper (https://doi.org/10.1073/pnas.1620290114): "however, MAPK [and PI3K] inhibition had only a marginal impact on survival in advanced CRC patients". Thus it seems unlikely that their observation will have any clinical consequences.
Further, it has been shown previously (https://doi.org/10.1073/pnas.1620290114) that mutation of APC directly and acutely affects YAP signaling (via src-family kinases). In addition, also other studies have established a clear positive link between Wnt and YAP signaling leading to cooperativity of these two pathways in colorectal tumorigenesis (reviewed here: doi: 10.1111/febs.15017). This may indicate that their TC4 cluster may simply derive from high Wnt activity in some of the mutant cells?
Their gradient of MAPK activity put the Wnt-high stem/TA cells at the end indicative of a negative impact of MAPK activity on the Wnt pathway. This has already been published (doi: 10.1242/dev.185678) and it was shown that ERK activity inhibits Wnt signalling while loss of Erk activity mediated Wnt stimulation. Thus also their TC1 cluster and its opposing positioning away from the Wnt high cluster is already known. Moreover, it had been shown in this earlier publication that gene ablation of Erk1/2 diminished goblet cell lineage specification in line with their observation that goblet like cells are at the end of their MAPK trajectories. Lastly, in MAPK wt tumors, it had been shown previously (DOI: 10.1038/s41467-018-04527-8) that activation of EGFR signalling is under the direct control of the Wnt signalling pathway by regulating expression of EGFR, Lrig3 and others. This indicates that intrinsic alterations of MAPK activity may be direct consequences of Wnt pathway activity.

Nevertheless, this manuscript is worth being published as the data presented in the paper are of great interest for the field and some of the observations might be relevant to the community. At the end, it's not an easy task to disentangle all the interconnections in pathway behaviour in such complicated system as CRC and any new piece of data can bring the community closer to answers.

Detailed comments:

The authors need to provide a list of genes which are characteristic for each of the TC1-4 clusters

they identified. There is some brief description for some of these clusters in the results, but this is not sufficient to understand the identity of these clusters.

Normal tissue contains few cells in clusters TC1 and TC2 indicating that these two phenotypes are not uniquely tumor specific. Strikingly, within the tumor all of the 4 TC clusters are strongly increased in copy number normal (CNN) cells indicating contribution of non-mutated cells to the tumor mass and responsiveness of these normal cells to potentially existing paracrine signals present in these tumors. In particular cluster TC2 has a large (about 30%) contribution of normal cells, but also clusters TC3 and TC4 are present in non-mutated cells in the tumor tissue in contrast to adjacent normal tissue which doesn't contain these phenotypes. The Authors should comment on this.

Organoid cultures were prepared from only 2 of these tumors (P009 and P013) and used for functional studies, which involves culture with Wnt/RSPO and EGF. Unfortunately, both of these tumor samples carry APC LOH while 30% of their samples i.e. P007, P012, P020, P026 are not mutant for APC. It would be essential to show that the conclusions they obtain from the functional studies of 2 APC mutant tumors also apply to these other tumors likely driven by other mechanisms.

For example, they show that removal of Wnt/RSPO does only weakly affect the cultures of P009/P013 (there is some reduction in Wnt pathway activity), but one wonders if this is still observed in the other, possibly not Wnt-driven samples.

Further, they always maintain addition of EGF to these cultures and then observe a gradient of MAPK activity. EGF is not required in these cultures of mutant organoids, so one wonders how these patterns look like when EGF is removed as well.

Moreover, it was not clearly specified in the manuscript what exact genes are included in this MAPK signatures. Are these strictly only downstream targets regulated specifically by MAPK signalling, or is it also components of the MAPK pathway, so that we may actually look at the cell's competence to respond to MAPK signals and not so much at actual signalling strength?

The paper would definitely benefit from including fluorescent ERK sensors (e.g. available from Addgene) in these organoids so that actual signalling strength can be measured on a per cell basis and cells can be sorted based on this activity followed by RNAseq to study how signalling strength compares to late effects on RNA expression.

Fig4A,B appears to have been generated from all CNA tumor cells of all patient samples. Sorting into TC1/4 high for MAPK high and TC3/Stem/TA high in MAPK low is basically a self-fulfilling prophecy since clusters TC1/4 are exactly characterized by a large number of MAPK targets. To me it is not clear if this really reflects graded signaling or simply the competence of cells to respond to MAPK signals (see above) and to what extent the results from RNA velocity are comparable to this sorting simply based on MAPK signatures.

In 4C it becomes evident that this sorting into TC1/4 high for MAPK high and TC3/Stem/TA high in MAPK low is observed more or less only in samples P009 and P013 (exactly the two samples they selected for organoid analysis, one wonders if this is by chance) while all other samples (in particular P008, P012, P016, P017, P020) don't show this pattern or simply lack the clusters (TC1, TC3, TC4, stem/TA) required to make this whole analysis (P007, P021, P025). This clearly shows that the link between graded MAPK target gene expression and differentiation states is clearly not an uniform

observation in all tumor samples. The authors should take this into account when they conclude their results and mention this discrepancy in the result part.

Moreover, the very clear separation into TC1/4 high for MAPK high and TC3/Stem/TA high in MAPK low is actually observed best when combining all tumor cells from all samples. However, in reality these cells never coexisted. In particular samples P017, P021 and P025 appear to contribute the stem/TA signal which is so nicely evident in 4B while the same samples basically lack TC1/4 clusters. It is therefore highly questionable if the suggested link between MAPK activity and differentiation actually really exists in even one of their tumor samples.

Figures EV4b and Fig. 6C. Both organoids are not mutant for BRAF/RAS but divergent trajectories upon drug treatment are present only in P009T organoid. Why?

Strangely, the same also applies for the other samples. Basically for all of the pairs they use for wt, RAS or BRAF mutant organoids only one of the samples shows a clear trajectory before and after drug treatment, the other sample looks much less convincing. It seems that the responses they observe are not very specific or not related to the mutational status. One starts to think that with some more organoid samples, their final conclusions could be very different. In other words, their sample size is low and the effects they describe are seen only in half of their samples. Thus, the functional data on EGFR/BRAF/RAS inhibitors is not very convincing and the authors should clearly state in the result section that divergent trajectories after drug treatment (RAS/RAF wt P009T, P013T organoids) are not replicated in all samples. Likewise, the initial trajectories (OT227, OT302) they describe are not replicated and this should be stated in the results. Further, the authors should try to add an explanation for that discrepancy. With the current data, it has to be clearly stated that those effects are not linked to the RAS or BRAF mutational status. Ideally, more organoid samples would be included to better support their claims.

Further, I am missing important controls for this experiments. Would the same differentiation changes be observed if the organoids are treated by other drugs such as chemotherapy or drugs which affect other signalling pathways such as AKT or mTOR. In other words, is the response the authors describe indeed MAPK driven or it can be any other signalling pathway?

Additional comments:
For the clustering of CNN and CNA cells which is shown in Fig. 1G, it was unclear if this is from just one tumor sample or combined from all samples? Since every tumor sample is quite different, the authors should show these clusters for all tumor samples separately as they have done in EV1 for normal tissue vs. tumor.

Why there are only 10 tumors in Fig.2A? The authors should also show the data for P014 and P026 samples.

In P012 tumors, hotspot mutations are not present, however P012 shows the highest Wnt target levels. Is there a link?

Throughout the paper, it is often not clear what samples are used for what analysis. The authors showed that tumor samples partially consist of non-mutated cells. Were these cells removed from subsequent analysis? Were all analysis where the tumor sample is not mentioned performed using all tumor samples or have there been selections of samples included in these combined tumor samples?

Likewise, in the reply to the reviewers the authors state that they have removed cell cycle related genes in some but not all of the analysis. Could this affect some of the phenotypes and links they describe? i.e. are the same correlations observed when these genes are included? Further, I couldn't identify in which analysis these cell cycle genes have been removed; this needs to be much clearer stated in the main text and the figure legends?

Why are there much less latent time points in Fig. 3F compare to Fig. 3E? This isn't obvious?

It is strange that in 4A, the expression of EphB2 and Lgr5 is over a more narrow range for the order along Lgr5 as compared to the order along MAPK. Why is this, shouldn't these expression values be more similar? The clear gradient in the MAPK ordered samples stems largely from the less compressed range of expression of these two markers.

For Fig. 4D authors should plot both TC1 and TC4, Lgr5 and Goblet cell signatures for both organoids.

Just a technical note, figures 3B/C are not jpg but some kind of dynamically generated images. This every time brings my computer to a halt when it needs to re-calculate these images. This is really annoying and absolutely not necessary. Please replace these dynamic images with simple jpg or png to avoid this.

Many supp tables are mentioned wrongly in the text (e.g. line 182, should be supp. table 2 instead of supp. table 1; line 120 - supp. table 4 instead of supp. table 2).

Supp table 2 doesn't contain P012 mutational data, this should be corrected.

We would like to thank the editor and the three previous referees for providing us guidance to re-consider the focus of our CRC single cell manuscript for the new submission. In this revision, we now performed new experiments and revised the manuscript according to the suggestions of the two remaining referees. We now

- Performed a comprehensive analysis on single cell validation data sets from Lee et al (2020) and Qian et al. (2020). We show that our model of six patient-overarching CRC cell clusters can be applied to other data sets.
- Performed several new cell culture experiments to show growth factor dependence and response to chemotherapy of the six organoid lines employed in the functional studies,
- Revised our analysis of dynamical cell velocity in organoids, and now always show all six organoid lines side-by-side in the main figures to avoid confusion.

The new data and analyses resulted in the revision of main figures 5 and 6, as well as many extended view and Appendix figures, in the addition of two new Appendix figures, and in extensive edits to the manuscript text that are listed below and can be tracked in the new manuscript file.

We believe that our conclusions are now much stronger, and that our model of patient-overarching CRC cell state clusters and the MAPK drive connecting them can provide an important contribution towards a single cell consensus model of CRC.

Find the point-to-point response to the reviewers below:

Referee #1 (Comments on Novelty/Model System for Author):

confusion between cell types within one organoid going into different differentiation states, versus these effects across patients.
diversity within organoid can be model artefact and patient signal cannot be determined in small study

We agree that our study, incorporating single cell data from 12 patients and 6 organoid lines, is not comprehensive enough to stratify patients, yet our analysis finds common transcriptome-based states between the patients, and we see a common MAPK drive in 5 out of 6 organoids. While the response to treatment is different in the organoids, we also find commonalities, and the heterogeneous response agrees with clinical reality.
When revising the manuscript, we understood that the presentation of the organoid data made it hard to clearly understand those commonalities. We now revised the data presentation in the main Figures 5 and 6 of the manuscript, so that it is now apparent that (i) 5 out of 6 organoid lines followed the same MAPK driven differentiation trajectory, (ii) MAPK inhibition induces plasticity overriding developmental trajectories, and (iii) organoids responded to MAPK-directed treatment by downregulating Goblet-like differentiation, and upregulating stem cell-related gene expression signatures, both programs marking ends of developmental trajectories.

For a further comprehensive understanding and description of CRC cell states in tumors, community efforts will be required to identify consensus CRC cell types in larger cohorts, similar to the consensus molecular subtypes. That said, the revision experiments presented here and comparison to published cohorts will hopefully convince the reviewer that our finding of CRC cell states organized along a MAPK axis are not model artifacts but features that exist in many or most CRC patients.

Referee #1 (Remarks for Author):

In line with initial reviewer 2, I still have concerns about the strong generalized statements on patient independent common epithelial cell types. Resolution, sample size, patient cohort, all may be impacting some of the initial findings that serve as a backbone many further statements

We agree that findings of our study can be impacted by resolution, patient cohort, sample size. We have added further analyses on already published data sets, and re-phrased parts of the manuscript, as outlined below.

We would like to emphasize that – in our opinion - the key finding of our study is not the exact number of epithelial cell state clusters, but that epithelial CRC cells form a rather continuous gradient of cell states along signaling gradients, and we hope that this is now clearer in the revised manuscript (see, for instance, changes in the first chapter of the discussion, lines 343 ff.).

The authors strongly state :
(i) cells from all twelve tumors form common clusters, in particular the TC1-4 clusters, suggesting strong commonalities between CRCs of different origins or progression histories.
(ii) Absorptive differentiation appears to be universally blocked despite different progression histories.
(iii) copy number changes appear to responsible for individual gene expression differences, see below.
(iv) CRC tumor cell types appear to cluster by MAPK strength in all tumors
And: "In summary, our analyses define normal stem/TA-like cells, immature goblet-like cells, and TC1-4 cells as six main patient-overarching clusters of CRC cells.
This overall 6 group structure is important as the next set of experiments ranks the groups in terms of MAPK activation and through interventions suggest that these different states are MAPK driven
This is an interesting concept, but to fit more with the current literature and guide the reader, it would be good to have more perspective on
- The 'validity' of the 6 groups. Please compare to the many other CRC single cell studies and their conclusion on number and type of patient independent signal
- The more is known about the T1-4groups, the more we can proceed in a focused way to understand them . Comparison to similarly generated subgroups described in other papers would be useful.

To address these concerns, we have re-analysed other published data sets (Results, p. 7, lines 174-178 and new Fig. EV3). We have performed the most in-depth analysis on a single data set of Belgian CRC patients from Lee et al., Nat. Genet. (2020), , doi.org/10.1038/s41588-020-0636-z, and Qian et al. Cell Research (2020) https://doi.org/10.1038/s41422-020-0355-0, two CRC single cell studies using a dataset consisting of 24 tissue samples (8 patients, termed KUL01-KUL31, are represented by 3 samples each of normal, tumor border and tumor center tissue).

We downloaded the publicly available raw data and performed a *de-novo* analysis using our analysis pipeline. This resulted in a data set of 17 794 epithelial cells, more than in the original study, as we employ a less stringent cut-off for % mt reads. Of these, 13 049 cells were from tumor center samples, 6930 from tumor border, and 5184 were derived from normal tissue (for cell numbers per sample, see new Figure EV3 A).

We next performed somatic copy number calling on the center and border tumor cells using InferCNV. Here is a heat map for the copy number calling for the Belgian patient data.

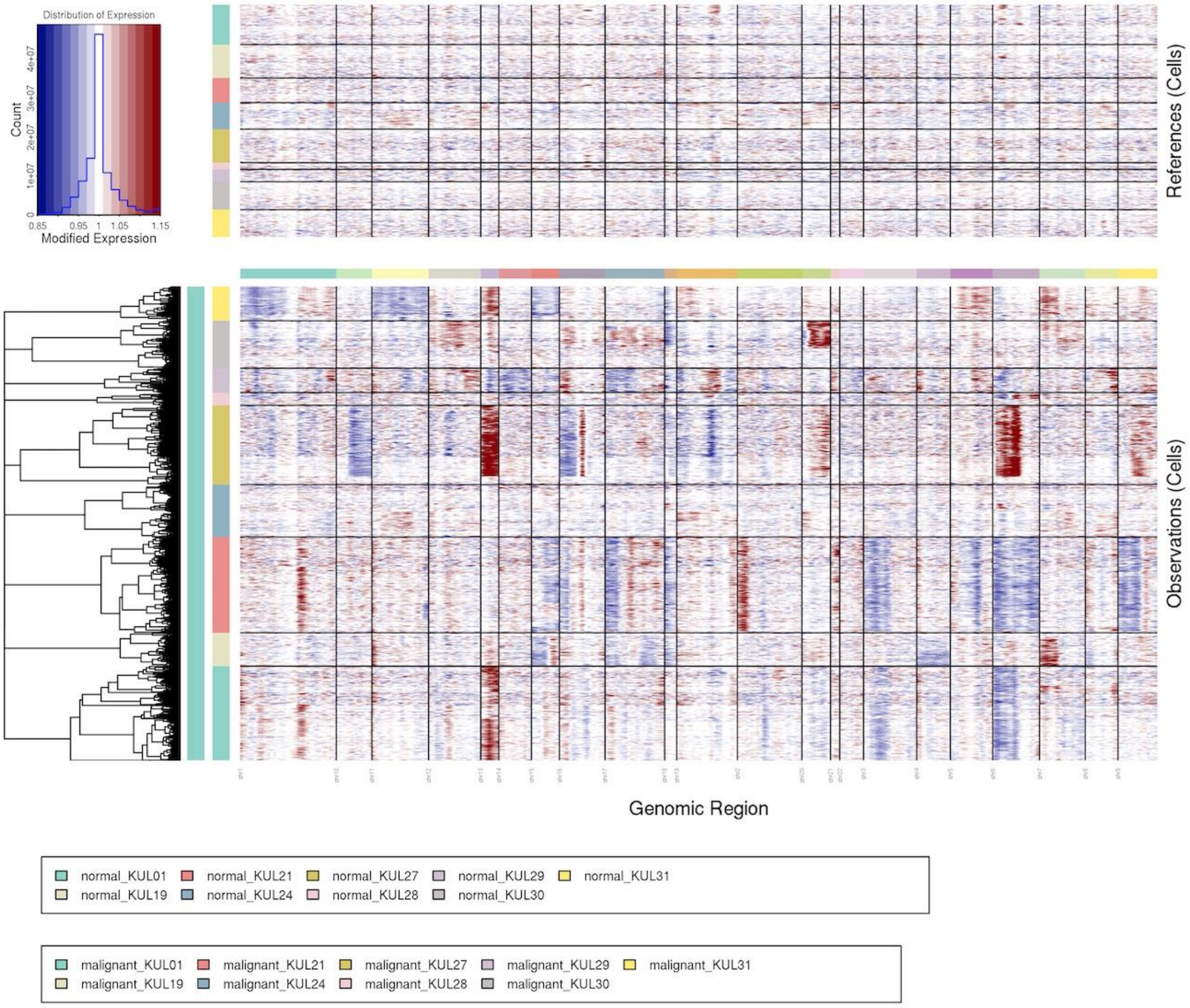

Referee Fig 1: Copy-number calling on data from Lee et al. (2020) and Qian et al. (2020). Top heatmap: reference dataset (normal epithelial cells from normal samples). Lower heatmap: epithelial cells from tumor border and center samples). Patient KUL24 was not used in the original papers, nor in our re-analysis.

It is of note that the original analysis from Lee et al. (2020) did not perform such an analysis and assumed all epithelial cells from tumor samples as tumor cells. We find that fractions of the epithelial cells in the Belgian patient samples are copy-number normal, in particular in the tumor border samples, though the fractions of CNN-epithelial cells is generally lower than in our patient cohort. A breakdown per sample of copy-number normal versus aberrant epithelial cells can be found in the new Fig EV 3B).

We next displayed the (CNA-)epithelial tumor cell data from Lee et al. as a UMAP. Using our preference of 10 PCA components, the epithelial cells intermingled. Using 50 components, the UMAPS separated by patients. This is similar to the observations we made for our own data (see main Fig 1C of our manuscript):

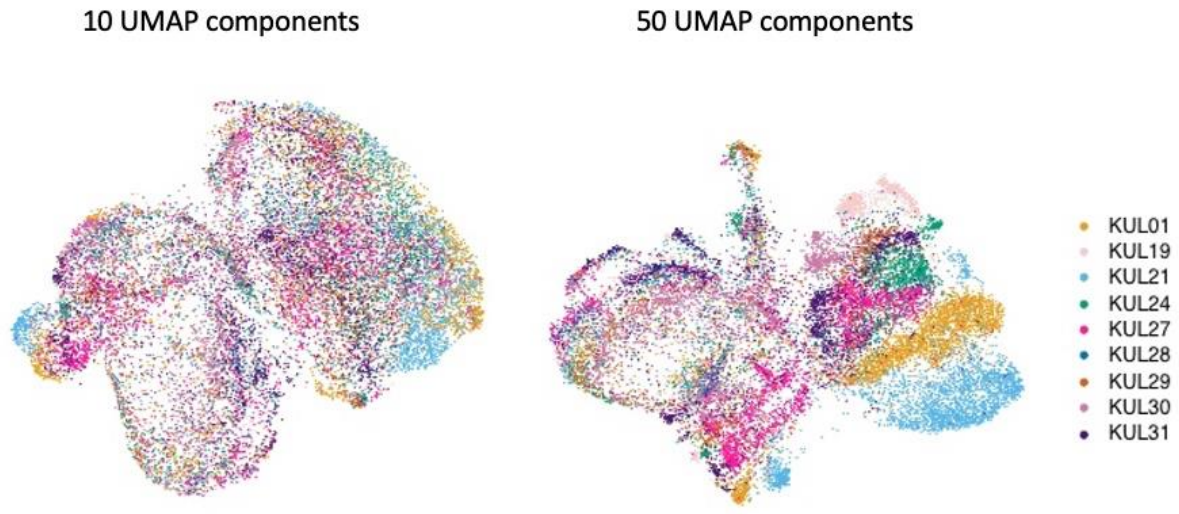

Referee Fig 2: UMAPs of filtered Belgian patient epithelial cell data from Lee et al. (2020) and Qian et al. (2020).

We next used the Ingest algorithm to superimpose the Belgian patient data into the gene expression space of our primary epithelial cell data essentially represented by the UMAPs in our main Fig. 1 (see Fig. EV 3C) and called cluster affiliation for each cell of the Belgian data set by nearest neighbor.

The resulting clusters of the Belgian patient CNA epithelial cells were next checked for signaling activities. Belgian patient transcriptomes assigned to the stem/TA, TC1-4 or Goblet-cell-like clusters (thus representing the six main cell state clusters in our manuscript) were compared side-by-side to our original data (Fig. EV 3D). We found that CNA tumor cells of clusters TC1 and TC4 were enriched in MAPK activity, while TC1 was enriched for the Hallmark DNA damage replicative stress signature and TC4 was high for the expression of YAP targets, in agreement with the pathway activity scores in our data set.

We next looked at cell type distributions in the Belgian patient data set (Fig. EV 3E-F). Cell type distributions of the KUL01-KUL31 patient dataset confirmed several of our key findings:

- We found the highest proportion of TC1-4 cells in the CNA tumor cells (50-60% for both tumor center and border), while the proportion was much lower for normal or CNN tumor sample (<20%) cells.
- We found a high proportion of enterocytes in the normal tissue and among CNN tumor cells, while these cells were present only at a smaller proportion in CNA tumor cell samples.
- We found that cells with a “normal stem cell signature” (the cluster termed “stem cells”, displayed in bright light blue) were present mainly in normal tissue, while a large proportion of cancer cells were assigned to other clusters of the stem cell/TA continuum.

Essentially, these distributions of cells in the Lee et al. (2020), Qian et al. (2020) validation data set are in line with our primary analysis, and we got similar results when performing the same analysis on the set of Korean patients from Lee et al. (2020) (patients SMC01-SMC25, Fig. EV3 G).

It is of note that Lee et al. (2020) also attempted to sub-cluster epithelial cancer cells of the Korean SMC dataset, resulting in a 9-cluster structure (Fig. 2d of Lee et al. (2020)). The 9 states were similar to stem/transient-amplifying cells (clusters 2,3,4,7,8,9) or Goblet cells (clusters

1,5,6), which is in general agreement to our 6 cluster model also consisting of stem, TA and goblet-like cell types. It would be interesting to dig deeper into overlaps of the Lee et al. (2020) 9 cluster model and our 6 cluster model; however the information on assignment of cells to the 9-cluster model of Lee et al. (2020), Fig 2d was not available to us.

We acknowledge that our analysis of the Lee et al. (2020) and Qian et al. (2020) data has limitations. In particular, our re-analysis relies on our original data structure. However, the analyses as performed for this revision show the existence of patient-overarching CRC cell states representing a continuum of signal pathway activities in the validation data set of eight Belgian patients, thus validating the main findings of our manuscript in an independent cohort.

An alternative approach would be to analyze a larger data set (such as pooled data from different studies) *de novo*. Such an analysis would probably be suited to uncover even more features of CRC cells, and would potentially also result in a cluster structure different from the 6 main CRC cell state clusters. However, such an approach of integrating independent third party data sets could also result in artifacts. Indeed, even the original Lee et al. (2020) paper never attempted to integrate the two original data sets of Belgian and Korean patients. We therefore suggest that integration of CRC single cell data sets of different origins is a more suitable task of a future community-wide consortium rather than an analysis that we can do in a revision, similar to the flurry of bulk transcriptome studies a couple of years ago that ultimately resulted in a consensus structure published as Guinney et al. Nat Med. 2015 doi: 10.1038/nm.3967. Also in this case, the original studies suggested data structures with different numbers of clusters, before a consensus was found. Importantly, such a consensus does not invalidate the original analyses performed by the different groups, but rather represents an agreement for future analyses. We envisage similar proceedings for CRC single cell data, and the contribution of our study is probably the focus on signaling pathway activities.

Ultimately, the analyses we performed in this revision show that third party data can also be assigned to our cell state model, resulting in clusters with differential pathway activities very similar to our original analysis.

- There are also premalignant polyp single cell data with patient independent groups and overlapping markers to those used here, would be good to add to the discussion

We have searched the literature for the data mentioned by reviewer #1, and found a preprint (Becker et al., 2021) that was only uploaded after submission of the review by reviewer #1: https://www.biorxiv.org/content/10.1101/2021.03.24.436532v1). We have added the manuscript to the discussion (Discussion, p. 13, line 361).

- The functional work in organoids here with MAPK inhibition and velocity to study causality has intrinsic value in establishing the concept of MAPK drive, but the more the paper can put the groups of cell types in perspective and increase confidence in their precise definitions, the more valuable the MAPK work becomes.

We think that we answered this remark already with the analyses presented above, but we would like to emphasize here that we already in the previous version of the manuscript tried to avoid the term “cell type” in favor of “cell state” to clearly highlight the transient nature of transcriptome states.

Line 83 add the relevant plasticity under therapy work of Lupo et al., Sci. Transl. Med. 12, eaax8313 (2020) 5 August 2020 . Line 286 and line 318 onwards ". Our findings thus indicate that

the MAPK, YAP and Wnt signaling pathways form an interconnected network controlling transcription, and ultimately proliferation and cell fate in CRC" please refer to the same paper, that already details this concept and incorporate their data in perspective here.

We thank the reviewer for drawing our attention to the excellent work of Barbara Lupo and colleagues and have now cited the work as suggested and re-phrased our paper. In the Lupo et al. study, the authors find upregulation of stem cell and secretory cell markers in residual tumor xenografts after anti EGFR therapy, and we believe our data is in agreement with the model proposed in Lupo et al.. However, the authors also state that stem and Paneth cell markers stain the same cell population, based on a few immunohistochemistry stainings that are hard to interpret (Lupe et al. Figure S6). In contrast, our data suggests that such cell populations can also be endpoints of different developmental trajectories (formerly Fig. 6D-H, now Fig. EV5 A-D).

Discussion line 358 ' that therapies targeting the MAPK pathway 359 reduce proliferation, but also redirect developmental trajectories of CRC cells towards goblet-cell 360 differentiation-like apoptotic or Wnt-driven stem-cell like endpoints that could be associated with 361 therapy-sensitivity or resistance, respectively. In summary, our analysis provides a single cell-based 362 framework for cell plasticity during cancer development and targeted therapy.' These broad statements are not very useful, certainly in light of

1. The very detailed work by Lupo mentioned above (which therapy in which patient will give what effect)
2. The small cohort here that cannot reliable detect patterns
3. The wild type versus mutation status not taken into account into such statements

As we have not formally looked into therapy resistance, but rather describe re-routing of trajectories under therapy, we have re-phrased this and many other statements in the manuscript. Furthermore, we fully agree that our cohort is small (similar to all other cohorts published so far) and that resistance mechanisms depend on patterns of predictive mutations and further yet unidentified features between CRCs that likely include CNA patterns, cell-of-origin epigenetic traits, and different (immune) microenvironments. We have now stated this in the discussion (p. 13, line 349, and in many small changes on page 14).

Referee #2 (Comments on Novelty/Model System for Author):

This manuscript uses single cell RNAseq of 12 tumor samples and some functional experiments with organoids (6 samples) to better characterize colorectal cancers. Their single cell RNAseq analysis of tumor material identified 6-7 cell phenotypes to be present in mutated (as measured by CNA) epithelial cells. This includes stem and TA cells, immature secretory (goblet) cells and four populations which are more tumor specific: TC1=cells in G2/S and with increased replication stress, TC2 = cells with high PI3K activity, T3=cells without mentioned signature, and TC4 = cells with high YAP target signature. Both, TC1 and TC4 show also high ERK/MAPK target signatures. The authors then continue to perform functional experiments with 2 organoid samples derived from these primary tumors and 4 other organoid samples described previously. They conclude that MAPK signalling affects the differentiation state in these tumors and that EGFR/MEK/BRAF inhibition can affect this.

Overall, the manuscript is largely descriptive, the functional data on EGFR/BRAF/RAS inhibitors is not convincing and has either to be expanded with more organoids or their claims have to be toned down.

Basically all of their observations have already been published. Their observation that MAPK inhibition can affect intestinal tumorigenesis is not novel and MEK as well as Erk inhibitors have already previously been shown to reduce tumor formation in APC mutants (https://doi.org/10.1111/cas.12670; DOI: 10.1038/nm.2143). But as Michael Karin has formulated it already in his paper (https://doi.org/10.1073/pnas.1620290114): "however, MAPK [and PI3K] inhibition had only a marginal impact on survival in advanced CRC patients". Thus it seems unlikely that their observation will have any clinical consequences.

Further, it has been shown previously (https://doi.org/10.1073/pnas.1620290114) that mutation of APC directly and acutely affects YAP signaling (via src-family kinases). In addition, also other studies have established a clear positive link between Wnt and YAP signaling leading to cooperativity of these two pathways in colorectal tumorigenesis (reviewed here: doi: 10.1111/febs.15017). This may indicate that their TC4 cluster may simply derive from high Wnt activity in some of the mutant cells?
Their gradient of MAPK activity put the Wnt-high stem/TA cells at the end indicative of a negative impact of MAPK activity on the Wnt pathway. This has already been published (doi: 10.1242/dev.185678) and it was shown that ERK activity inhibits Wnt signalling while loss of Erk activity mediated Wnt stimulation. Thus also their TC1 cluster and its opposing positioning away from the Wnt high cluster is already known. Moreover, it had been shown in this earlier publication that gene ablation of Erk1/2 diminished goblet cell lineage specification in line with their observation that goblet like cells are at the end of their MAPK trajectories. Lastly, in MAPK wt tumors, it had been shown previously (DOI: 10.1038/s41467-018-04527-8) that activation of EGFR signalling is under the direct control of the Wnt signalling pathway by regulating expression of EGFR, Lrig3 and others. This indicates that intrinsic alterations of MAPK activity may be direct consequences of Wnt pathway activity.

Nevertheless, this manuscript is worth being published as the data presented in the paper are of great interest for the field and some of the observations might be relevant to the community. At the end, it's not an easy task to disentangle all the interconnections in pathway behaviour in such complicated system as CRC and any new piece of data can bring the community closer to answers.-

We thank the reviewer for stating that our manuscript is worth of being published, but we disagree with many other statements in the “Comments on novelty/Model system” section.

In general, we are aware of a large body of literature dealing with the Wnt, MAPK and YAP pathways in colorectal cancer. However, this does not mean that “basically all of (our) observations have already been published”, as the referee states.

For instance, the referee correctly states that our observation that MAPK inhibition can inhibit colorectal tumorigenesis is not novel. Of course we are aware of this, as EGFR-MAPK inhibition is first-line therapy for mCRC patients without KRAS/NRAS/BRAF mutations in Europe, America, and probably also elsewhere, and this medical guideline is based on decades of research of MAPK signaling in CRC. In this context, we wonder how the publication cited by the author (Fujishita et al., Cancer Science 2015) about a stromal COX-2 effect after MEK inhibition in APC-mutant mice is relevant for this point. We were also puzzled about the quote from a paper by Michael Karen that appeared to dismiss the great medical relevance of MAPK inhibition in CRC. We therefore looked up the paper and found that the referee used the quote out of context, as it originally referred to the minor clinical advantage of a dual targeting strategy involving PI3K and MAPK signaling, a subject not touched by our work.

Likewise, we are aware that interactions of Wnt and MAPK pathways are well studied in the intestinal epithelium and in CRC. We could look back to Phelps et al. Cell (2009) DOI: 10.1016/j.cell.2009.02.037, and consider the case closed. But, of course, dozens or even hundreds of papers have contributed incremental improvements of our understanding since then. As the referee correctly states, Wei et al. have published in Development (2020) “that ERK activity inhibits Wnt signaling” in the intestinal epithelium among other findings, based on ERK knockout mouse models. For the record, we have reached the same conclusion using transgenic overexpression of oncogenic BRAF in the mouse a couple of years earlier in Riemer et al., Oncogene (2015). However, Wei et al. did neither study CRC nor cell trajectories. Thus, we think that many future studies, including ours, can add to our understanding of Wnt and MAPK signaling and cell development in CRC.

Detailed comments:

The authors need to provide a list of genes which are characteristic for each of the TC1-4 clusters they identified. There is some brief description for some of these clusters in the results, but this is not sufficient to understand the identity of these clusters.

The list of genes that the referee is looking for was provided as Supplementary Table 4. We think that the best characterization of the clusters is by their inferred pathway activities, as shown in Fig. 2A.

Normal tissue contains few cells in clusters TC1 and TC2 indicating that these two phenotypes are not uniquely tumor specific. Strikingly, within the tumor all of the 4 TC clusters are strongly increased in copy number normal (CNN) cells indicating contribution of non-mutated cells to the tumor mass and responsiveness of these normal cells to potentially existing paracrine signals present in these tumors. In particular cluster TC2 has a large (about 30%) contribution of normal cells, but also clusters TC3 and TC4 are present in non-mutated cells in the tumor tissue in contrast to adjacent normal tissue which doesn't contain these phenotypes. The Authors should comment on this.

We thank the reviewer for pointing out this interesting aspect of our data. As part of the revision analyses, we performed a validation analysis using an independent single cell CRC data set, comprising of samples from normal, tumor border and central tumor tissue (discussed in depth above, response to rev#1). In agreement with our own data, we find that some TC cell types, such as TC1, are also present to a small extent in normal tissue, while others, such as TC3, are represented also in copy-number (normal) epithelial cells derived from tumor samples.

We therefore think that the reviewer is right to assume that part of the gene expression patterns characteristic for the TC1-4 cell states (related to cell signaling activities, see main Fig 2A) can be induced not only by oncogenic mutations, but probably also by paracrine signaling in the normal or tumor microenvironments. Presence of oncogenic mutations could thus increase the likelihood of transcriptomes to assume a TC1-4 state.

As TC1-4 content is still much higher in CNA tumor cells across datasets, we would like to suggest that oncogenic mutations and paracrine signaling possibly co-operate in inducing gene expression changes characteristic for TC1-4 cells (e.g. MAPK can be activated by mutations in RAS/RAF genes, but also by production of receptor tyrosine kinase ligands at the invasive front). We have discussed this now on p. 13, lines 364-368 of the revised manuscript.

Organoid cultures were prepared from only 2 of these tumors (P009 and P013) and used for functional studies, which involves culture with Wnt/RSPO and EGF. Unfortunately, both of these tumor samples carry APC LOH while 30% of their samples i.e. P007, P012, P020, P026 are not mutant for APC. It would be essential to show that the conclusions they obtain from the functional studies of 2 APC mutant tumors also apply to these other tumors likely driven by other mechanisms.

Unfortunately, we have not succeeded in generating organoids from other tumors of our study cohort, as organoid generation is secondary to the single cell workflow and depends on a couple of free extra hands and enough cells. However, we agree that analysis of organoids with diverse genetics is important and had, therefore, already added four further lines with KRAS or BRAF mutations during the previous revision, and now added further analyses and performed more experiments, as outlined below. The two BRAF-mutant lines are APC-wildtype and data on these organoids was already present in the previous revision, e.g. we already showed development along a MAPK gradient for these models (see Fig. 6 A, previously Fig EV4, now with additional data and statistics in Table EV6, as outlined below).

For example, they show that removal of Wnt/RSPO does only weakly affect the cultures of P009/P013 (there is some reduction in Wnt pathway activity), but one wonders if this is still observed in the other, possibly not Wnt-driven samples.

There is considerable evidence that CRCs originating via non-conventional progression pathways (such as the serrated pathway) ultimately also become intrinsically Wnt/beta-catenin-active (that is, independent from extrinsic Wnt sources) by mechanisms unrelated to APC loss (for data on mouse models, see Rad et al. Cancer Cell. 2013 doi: 10.1016/j.ccr.2013.05.014, for human data see Yachida et al. Am J Surg Pathol. 2009 Dec;33(12):1823–32 or Wu et al., Am J Clin Pathol. 2008 Mar;129(3):416–23).

To formally test whether the lines used in our study are Wnt-dependent, we now cultured all six organoid lines in the presence vs absence of Wnt/R-Spondin. We find that all lines can grow in the absence of Wnt/R-Spondin, but that the BRAF-mutant line B2040 profits from addition of Wnt and Rspondin to the medium. The data are now given as part of the new Appendix Fig. S6.

Further, they always maintain addition of EGF to these cultures and then observe a gradient of MAPK activity. EGF is not required in these cultures of mutant organoids, so one wonders how these patterns look like when EGF is removed as well.

We do not know the basis for the referee's assumption that our KRAS/BRAF mutant organoid cultures do not require EGF, as we had not formally tested this. However, the reviewer is correct in the interpretation of our workflow, that we maintain culture medium including EGF throughout our MAPK therapy experiments (Fig. 5, 6), as also stated in the methods section.

We now also tested whether EGF is required for growth of the CRC organoid lines employed, and expanded the aforementioned new Appendix Figure S6 to two further conditions, that is, minus EGF (basal medium only), and addition of an EGFR inhibitor, to in addition inactivate potential signals by EGFR ligands present in the Matrigel. As expected, the response of the cultures to EGF addition/deprivation is heterogeneous: the KRAS-mutant line OT227 and the BRAF-mutant line C2019 appear to be EGF dependent for effective growth, while the KRAS-mutant line OT302 and the BRAF-mutant line B2040 are not overtly EGF-dependent.

In summary, in light of the heterogeneous response of the CRC organoid lines to EGF and/or Wnt/Rspo, we maintain that our approach of using standardized media for the organoids experiments is justified, as now also clearly stated in the results part, page 10, lines 260-262.

Moreover, it was not clearly specified in the manuscript what exact genes are included in this MAPK signatures. Are these strictly only downstream targets regulated specifically by MAPK signalling, or is it also components of the MAPK pathway, so that we may actually look at the cell's competence to respond to MAPK signals and not so much at actual signalling strength?

The citation for the MAPK target gene signature used in this study was already given in the manuscript: M. Schubert B. Klinger M. Klünemann A. Sieber F. Uhlitz S. Sauer M.J. Garnett N. Blüthgen J. Saez-Rodriguez, Nat. Communications 9 (1): 20 (results, page 6, line 155). This signature includes only target genes, not pathway components. Indeed, one of the last authors of this study (NB) has been a driving force in rigorously defining cancer-related pathway target gene signatures for the community in many frequently-cited studies.

The paper would definitely benefit from including fluorescent ERK sensors (e.g. available from Addgene) in these organoids so that actual signalling strength can be measured on a per cell basis and cells can be sorted based on this activity followed by RNAseq to study how signalling strength compares to late effects on RNA expression.

We agree that fluorescent ERK sensors are great tools. We have, therefore, used such sensors already in our previous publication Brandt and Sell et al., Nat. Commun. 2019 doi.org/10.1038/s41467-019-10954-y, to do exactly what the reviewer suggests: measure signalling strength on a per cell basis, sort cells based on this activity, and study how signalling strength compares to effects on RNA expression.

In addition, other groups have published extended ERK signalling studies using different fluorescent sensors (Gillies et al, Cell Systems 2017, doi.org/10.1016/j.cels.2017.10.019, Ponsioen et al Nat Cell Biol (2021). doi.org/10.1038/s41556-021-00654-5).

Here, we have used CyTOF analysis to measure ERK phosphorylation on a cell-to-cell basis as part of our previous revision. We believe that using fluorescent ERK sensors as part of this new revision is clearly beyond the scope of this study.

Fig4A,B appears to have been generated from all CNA tumor cells of all patient samples. Sorting into TC1/4 high for MAPK high and TC3/Stem/TA high in MAPK low is basically a self-fulfilling prophecy since clusters TC1/4 are exactly characterized by a large number of MAPK targets. To me it is not clear if this really reflects graded signaling or simply the competence of cells to respond to MAPK signals (see above) and to what extent the results from RNA velocity are comparable to this sorting simply based on MAPK signatures.

The reviewer is right that Fig 4A-C was generated from CNA tumor cells, as was already stated in the manuscript (page 8, line 224: "We assigned the SCN-aberrant primary cancer cell transcriptomes to 40 bins along a gradient…) Indeed, TC1 and TC4 contain many cells with a high MAPK signature and thus they are found predominantly to the left when sorting for MAPK. Insofar, Figures 2 and 4 are complementary. However, we would not call this a "self-fulfilling prophecy", as we did not produceTC1/4 by setting aside MAPK-high cells, but the clusters formed on the k nearest neighbor graph, a computational process that does not know about MAPK target gradients, unless these form a major component of the covariance in the data.

The reviewer also raises an interesting point by asking why the TC1/4 cells are MAPK-high. Is this intrinsic competence? Is it paracrine signaling? We do not know for sure at this point, but our previous work (Brandt, Sell et al., (2019), see above and discussion) establishes that intestinal epithelial and CRC cells can assume states with different intrinsic competence to respond to EGFR/RAS signals.

In 4C it becomes evident that this sorting into TC1/4 high for MAPK high and TC3/Stem/TA high in MAPK low is observed more or less only in samples P009 and P013 (exactly the two samples they selected for organoid analysis, one wonders if this is by chance) while all other samples (in particular P008, P012, P016, P017, P020) don't show this pattern or simply lack the clusters (TC1, TC3, TC4, stem/TA) required to make this whole analysis (P007, P021, P025). This clearly shows that the link between graded MAPK target gene expression and differentiation states is clearly not an uniform observation in all tumor samples. The authors should take this into account when they conclude their results and mention this discrepancy in the result part.

Moreover, the very clear separation into TC1/4 high for MAPK high and TC3/Stem/TA high in MAPK low is actually observed best when combining all tumor cells from all samples. However, in reality these cells never coexisted. In particular samples P017, P021 and P025 appear to contribute the stem/TA signal which is so nicely evident in 4B while the same samples basically lack TC1/4 clusters. It is therefore highly questionable if the suggested link between MAPK activity and differentiation actually really exists in even one of their tumor samples.

We disagree with these statements. To address the referees' point, we show here that the cluster representation is graded along the MAPK axis in individual patients. We do this by binning the CNA tumor cells (as shown in Fig 4c) along the MAPK axis per patient (or in the words of the reviewer: We bin only cells that "in reality ... coexisted"). We find that cell state representation is clearly uneven across the MAPK gradient not only in patients P009 and P0013, but also in the other patients. Cell gradients usually conform to our general model (TC1, 4 = MAPK high; Goblet-like, stem cell-like = MAPK lower). The finding extends to the samples that the reviewer picks as examples to the contrary: P007 has statistically significant uneven representation of all TC cell states along the MAPK gradient with TC4 showing the strongest gradient. Likewise, TC4 cells are

also represented in the MAPK-high bins in P008, and a larger representation of goblet-like cells in the MAPK-lower bins, similar to P012 where the MAPK-lower bins are populated by stem/TA-like cells etc. Statistical tests are given as as a new Table EV6.

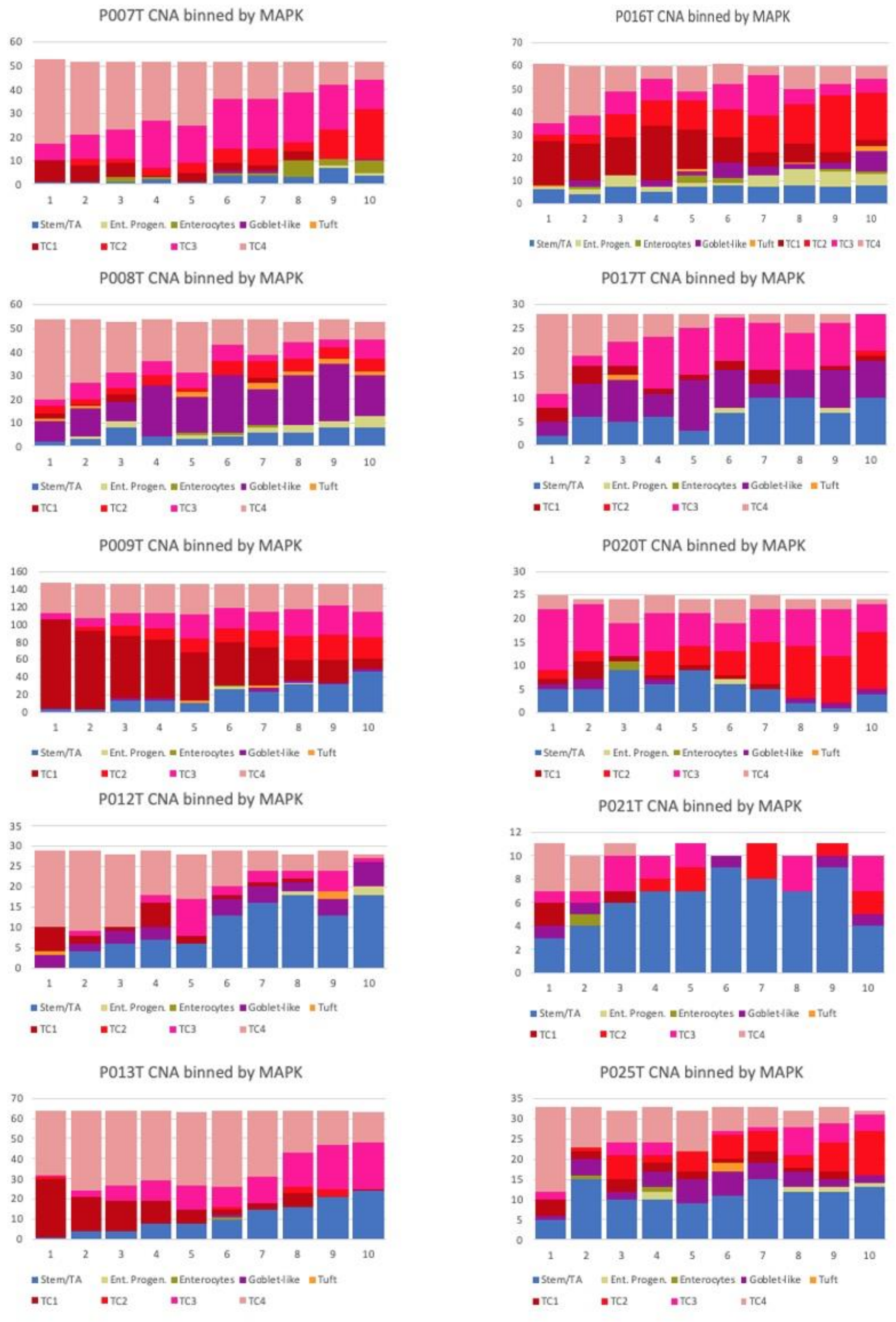

Referee Fig.3: Cell state distribution across the patient-specific MAPK axis (SCN-aberrant tumor cells ordered into 10 bins per patient, high to low MAPK)

Figures EV4b and Fig. 6C. Both organoids are not mutant for BRAF/RAS but divergent trajectories upon drug treatment are present only in P009T organoid. Why?

As already stated in response to the last two points raised by this reviewer, we do not expect that CRC cells from different tumors behave the same, even if they share predictive mutations. So, again, it is impossible to answer such a question. Why does tumor heterogeneity, and therefore heterogeneity in organoid response, exist? Differences in somatic mutations or copy number

changes, epigenetic cell-of-origin differences, differences in patient genomes etc. will all play roles here. We do not state that divergent trajectories are a universal feature of plasticity after MAPK-directed therapy – but we observe them in some of the models we employed in this study.

Strangely, the same also applies for the other samples. Basically for all of the pairs they use for wt, RAS or BRAF mutant organoids only one of the samples shows a clear trajectory before and after drug treatment, the other sample looks much less convincing. It seems that the responses they observe are not very specific or not related to the mutational status. One starts to think that with some more organoid samples, their final conclusions could be very different. In other words, their sample size is low and the effects they describe are seen only in half of their samples. Thus, the functional data on EGFR/BRAF/RAS inhibitors is not very convincing and the authors should clearly state in the result section that divergent trajectories after drug treatment (RAS/RAF wt P009T, P013T organoids) are not replicated in all samples. Likewise, the initial trajectories (OT227, OT302) they describe are not replicated and this should be stated in the results. Further, the authors should try to add an explanation for that discrepancy. With the current data, it has to be clearly stated that those effects are not linked to the RAS or BRAF mutational status. Ideally, more organoid samples would be included to better support their claims.

We disagree with these statements, but we understand that the presentation in the previous manuscript made it hard to disentangle common and divergent behavior between the organoid lines. We have re-organized the presentation (Fig. 5, 6, results, p.9-12) so that it is more obvious that:

(1) Signaling responses to inhibitor treatment are, as expected, different between different classes of organoids, but similar within these classes (RAS/RAF wt, RAS mut, BRAF mut). (Fig. 5 C,D, Fig. 6C)
(2) RNA velocity analysis shows clear developmental trajectory in all organoids except one that lead from MAPK-high to MAPK-low cells under control conditions, replicating and extending our initial finding for the P009T and P013T lines in 5/6 lines (Fig. 6A).
(3) All organoids respond to an effective MAPK inhibition ("preferred treatment") by downregulating differentiation-associated Goblet markers or upregulating Wnt-driven stemness markers or both, leading to trajectories with different endpoints (Fig. 6D-F).

Similar to the points raised above, we do not think that it is unexpected that different models show heterogeneous response, as CRC is a heterogeneous disease. Here, the reviewer states that responses of our drug assays are unrelated to the RAS/RAF mutational status, however, this is not what our data shows. There are clear similarities between the responses of the two RAS/RAF-wt organoids, the two KRAS-mutant lines and the two BRAF-mutant lines, as shown in our analysis of signaling networks (Fig. 5C), and transcriptomes Fig. 5D, Fig. 6A,B), and described in the respective part of the results section (eg page 10, lines 269 and following, page 11, 301 and following). But of course there are also differences between the lines. These contribute to differences in trajectories, which should not be assessed in isolation.

The reviewer states that our initial finding of MAPK gradients along the trajectory was not replicated by the new experiments added during the previous revision, but this is not true. Indeed, we were clearly able to **replicate** the MAPK gradient first shown in Fig. 2D along latent time in the new experiments with the P009T and P013T lines. We have now also added statistics for the figure to make the point more obvious (Table EV6). Furthermore, we could also **extend** our findings, as also three of the four new lines show a statistically significant MAPK gradient along latent time (see Fig. 6A). In summary, we see a MAPK gradient along latent time in five out of six lines (Fig. EV4 A). The sixth line (OT227) has no clear developmental gradient under the culture

conditions used, see also the lack of dynamical velocity in DMSO condition, EV4 C. Cell plasticity is induced by anti-MAPK therapy in all six lines, albeit in different ways (but generally in line with our model, see signature data in Fig. 5D, in conjunction with the treatment responses quantified in Fig. 6B).

Further, I am missing important controls for this experiments. Would the same differentiation changes be observed if the organoids are treated by other drugs such as chemotherapy or drugs which affect other signalling pathways such as AKT or mTOR. In other words, is the response the authors describe indeed MAPK driven or it can be any other signalling pathway?

It is indeed an interesting question if the observed induced phenotypes are specific for MAPK blockade, or induced by cytotoxic drugs in general. In an initial test we used OT227 and OT302 to determine chemosensitivity. We could clearly see phenotypic effects between 1uM and 10uM of 5FU or Oxaliplatin, as exemplified here:

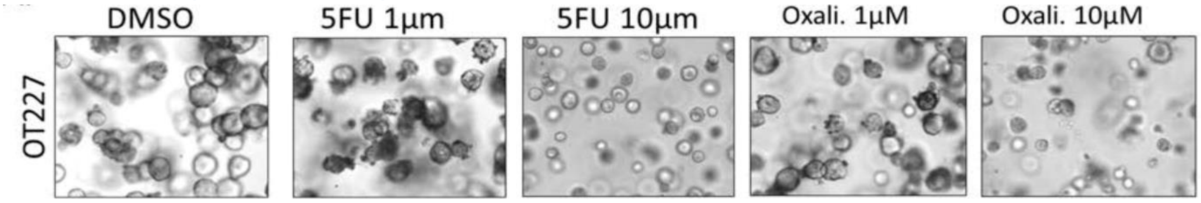

Referee Fig. 4: Phenotypic effects of chemotherapy on OT227 organoids

We next treated all six cultures with 5uM of either substance for 48h, in addition of replicating the shortlisted anti-MAPK treatment for the individual lines. We see that the effects of chemotherapy are clearly different from anti-MAPK therapy on the level of the single cell transcriptome. Also, cell cycle distribution is differentially affected. While MAPK inhibition generally resulted in G1 arrest, 5FU-sensitive lines, such as OT227 and P009T, showed a G2M arrest, in agreement with DNA damage response. These data are now the new Appendix Fig. S8, and are referenced from the main results part, line 309-311.

Additional comments:

For the clustering of CNN and CNA cells which is shown in Fig. 1G, it was unclear if this is from just one tumor sample or combined from all samples? Since every tumor sample is quite different, the authors should show these clusters for all tumor samples separately as they have done in EV1 for normal tissue vs. tumor.

Fig 1G is the combined UMAP for all tumor epithelial cells, and we have added the information to the figure legend. We have added the requested panels (CNA/CNN) for patients separately as revised Fig. EV 2.

Why there are only 10 tumors in Fig.2A? The authors should also show the data for P014 and P026 samples.

As we restrict the analyses to CNA tumor cells, we can show analyses only for the 10 out of 12 tumors where we find CNA cells (see results, page 6. Lines 133 ff: "*We identified clusters of SCN-aberrant epithelial cells in ten out of the twelve tumors (Fig. 1F, Supplementary Fig. 5A). Exome sequencing of tumors P007, P008 and P009 validated SCNA calling from transcriptomes,*

showing that the procedure is robust for our single cell data (Supplementary Fig. 5B). P014 and P026 contained no cells with overt SCNAs. This was expected for tumor P026, which is MSI and thus defined by single nucleotide polymorphisms rather than SCNAs, but unexpected for P014, which was diagnosed as BRAF-mutant however MSS.")

In P012 tumors, hotspot mutations are not present, however P012 shows the highest Wnt target levels. Is there a link?

We unfortunately could not determine the hotspot mutational pattern for P012, as we could not sequence tumor P012 due to shortage of material (see also below). As all other available material was reserved for patient diagnosis, we cannot overcome this limitation.

Throughout the paper, it is often not clear what samples are used for what analysis. The authors showed that tumor samples partially consist of non-mutated cells. Were these cells removed from subsequent analysis? Were all analysis where the tumor sample is not mentioned performed using all tumor samples or have there been selections of samples included in these combined tumor samples?

After we break down tumor epithelial cells by copy number status (Fig. 1F,G) we restrict further analyses (Figures 2 and 4) to CNA-tumor cells only. We have added further statements to the results part (p.6, eg line 153) to avoid confusion. We never select any subsamples for the analyses.

Likewise, in the reply to the reviewers the authors state that they have removed cell cycle related genes in some but not all of the analysis. Could this affect some of the phenotypes and links they describe? i.e. are the same correlations observed when these genes areM included? Further, I couldn't identify in which analysis these cell cycle genes have been removed; this needs to be much clearer stated in the main text and the figure legends?

The cell cycle was controlled for in all primary data and organoid analyses, by linear regression, as can be seen from the code. This was also explicitly mentioned in the Methods part on Organoid cell analysis, but we now also mention this in the chapter for the primary cell data analysis (methods part, p. 18, lines 500 ff).
To be clear, we did not remove cell cycle-related genes, but we used linear regression. That is, in order to focus any analysis on more subtle effects, we adjust for the strong effect of the cell cycle on each gene's expression. We first score the extent of expression of genes marking each cell cycle using gene set expression scoring. The difference between cycling and non-cycling scores against the expression of each gene separately is linearly regressed out. This procedure is standard and current best practice in the field, see Luecken et al., Mol. Sys. Biol. (2019), doi.org/10.15252/msb.20188746.

Why are there much less latent time points in Fig. 3F compare to Fig. 3E? This isn't obvious?

This is a matter of binning. As Single gene data (3F) is sparser and more noisy compared to signature scores, we had chosen wider bins for 3F. Both, 3E and 3F, cover the complete latent time scale. The same is true for the new Figure 6A.

It is strange that in 4A, the expression of EphB2 and Lgr5 is over a more narrow range for the order along Lgr5 as compared to the order along MAPK. Why is this, shouldn't these expression values be more similar? The clear gradient in the MAPK ordered samples stems largely from the less compressed range of expression of these two markers.

It is natural that by aggregating expression values from many cells into 40 bins, average expression of markers within the bins varies. Still, the basis is the same primary data. We use no compression in either subfigure. The LGR5-ISC is particularly suited to identify the very high LGR5-expressing cells, resulting in a greater bin difference between the first two and the subsequent bins. We have now mentioned this in the results part, p. 9, line 228.

For Fig. 4D authors should plot both TC1 and TC4, Lgr5 and Goblet cell signatures for both organoids.

We have added new panels showing all cell type and pathway signatures for all six organoid lines used in the functional assays, new Supplementary Fig. 7.

Just a technical note, figures 3B/C are not jpg but some kind of dynamically generated images. This every time brings my computer to a halt when it needs to re-calculate these images. This is really annoying and absolutely not necessary. Please replace these dynamic images with simple jpg or png to avoid this.

We now use Jpgs, which may however result in lower image quality.

Many supp tables are mentioned wrongly in the text (e.g. line 182, should be supp. table 2 instead of supp. table 1; line 120 - supp. table 4 instead of supp. table 2).

We thank the reviewer for pointing out the mistakes and have corrected them.

Supp table 2 doesn't contain P012 mutational data, this should be corrected.

No material for sequencing was available for this tumor. We have added this information as a note to Suppl. Table 2.

20th Jul 2021

Dear Dr. Morkel,

Thank you for the submission of your revised manuscript to EMBO Molecular Medicine. I am pleased to inform you that we will be able to accept your manuscript pending the following final amendments:

1) Please address all the points raised by the referee #1 and #2.
2) In the main manuscript file, please do the following:
- Correct/answer the track changes suggested by our data editors by working from the attached document.
- Remove text highlight colour.
- Add callouts for Figure EV2.
- Make sure that all special characters display well.
- Remove appendix figure legends.
- In M&M, Include a statement that, in addition to informed consent, the experiments conformed to the WMA Declaration of Helsinki and to the principles set out in the Department of Health and Human Services Belmont Report.
- In M&M, add statistical paragraph that should reflect all information that you have filled in the Authors Checklist, especially regarding randomization, blinding, replication etc.
- In addition to the accession number please provide URL for all deposited data. Use the following format to report the accession number of your data:

The datasets produced in this study are available in the following databases:
[data type]: [full name of the resource] [accession number/identifier] ([doi or URL or identifiers.org/DATABASE:ACCESSION])

Please check "Author Guidelines" for more information.
https://www.embopress.org/page/journal/17574684/authorguide#availabilityofpublishedmaterial
3) Tables: Move EV table legends from the main text file to the corresponding table file.
4) Source data: We encourage you to include the source data for figure panels that show essential data. Numerical data should be provided as individual .xls or .csv files (including a tab describing the data). For blots or microscopy, uncropped images should be submitted (using a zip archive if multiple images need to be supplied for one panel). Please check "Author Guidelines" for more information. https://www.embopress.org/page/journal/17574684/authorguide#sourcedata
5) Synopsis:
- Synopsis text: Please provide a short stand first (maximum of 300 characters, including space) as well as 2-5 one sentence bullet points that summarise the paper. Please write the bullet points to summarise the key NEW findings. They should be designed to be complementary to the abstract - i.e. not repeat the same text. We encourage inclusion of key acronyms and quantitative information (maximum of 30 words / bullet point). Please use the passive voice.
- Synopsis image: Please check your synopsis image, revise it if necessary and submit the final versions with your revised manuscript. Please be aware that in the proof stage minor corrections only are allowed (e.g., typos).
6) For more information: There is space at the end of each article to list relevant web links for further consultation by our readers. Could you identify some relevant ones and provide such information as well? Some examples are patient associations, relevant databases,

OMIM/proteins/genes links, author's websites, etc...
7) As part of the EMBO Publications transparent editorial process initiative (see our Editorial at http://embomolmed.embopress.org/content/2/9/329), EMBO Molecular Medicine will publish online a Review Process File (RPF) to accompany accepted manuscripts. This file will be published in conjunction with your paper and will include the anonymous referee reports, your point-by-point response and all pertinent correspondence relating to the manuscript. Let us know whether you agree with the publication of the RPF and as here, if you want to remove or not any figures from it prior to publication. Please note that the Authors checklist will be published at the end of the RPF.
8) Please provide a point-by-point letter INCLUDING my comments as well as the reviewer's reports and your detailed responses (as Word file).

I look forward to reading a new revised version of your manuscript as soon as possible.

Yours sincerely,

Zeljko Durdevic

Zeljko Durdevic
Editor
EMBO Molecular Medicine

***** Reviewer's comments *****

Referee #1 (Comments on Novelty/Model System for Author):

organoids were not generated for all models, so matching single cell observations with functional testing not possible for all

Referee #1 (Remarks for Author):

There have been significant improvements in clarity, data density and data presentation in the manuscript.
As a resource the manuscript is certainly worth publishing, and at minimum generates a well developped hypothesis.
A lot of effort hab been put into the manuscript for which we commend the authors. However the findings remain hypothesis generating at best and cannot be considered validated or netirely accurate in it's present form. This however is possibly beyond the scope of this manuscript. If sufficient 'warning and context ' language is used, the manuscript can be correcltly used as a data rich basis for further validation of the concept.
As pointed out in the point by point reply page 5, the 6 clusters are possibly not the final CRC structure and applying them in a supervised way to external datasets does not constitute

validation. Many other papers are published that stop at this level of validation indeed. What is more difficult are the conclusions relating MAPK gradients to the 6 clusters, if they are not stable then same for the rest...
Remarks of all revieuwers where in the same direction (see page 11 point by point section)and some of theselimitations should be highlighted.
Last there is the point of what is tumorcell with CNV, without CNV or contaminating normal. From the point by point document it is clear that this is yet unresolved. An arguments is made that tumor cells may exist without genomic abnormalities and be paracrine driven. all fine, but the number of NVC number cells is quite high for some patients. However all downstream analysis used only CNV abberrant single cell data, removing all tumor biology of CNV stable cells in the disovery and conclusions. The bias that this introduces is unknown.
I suggest to highlight many of these possible biases in the manuscript. There are some well elaborated replies in the point by point document that aknowledge gaps , I suggest to incorporate these to drive also further work by the community in taking the next steps based on this work

Referee #2 (Remarks for Author):

The authors have largely answered to my questions. There a re a few points which remain:

The authors have now added a part to the discussion that some of the TC1-4 cell phenotypes can be identified in CNN tumor cells and in normal adjacent tissue. However, the result text is still misleading regarding this interpretation of the data: it is still stated that "TC1-4 cells that have no normal tissue counterparts". This needs to be corrected.

It wasn't clear (or at least I couldn't find it) if the inhibitor treatments (BRAFi, MEKi, EGFRi) actually led to a block in organoid growth and massive cell death or just slowed down the cultures? Can you please add that information (e.g. survival curves) to the results?

Since this wasn't clearly spelled out in the main text, please add to the results that OT227 and OT302 are mutant for APC (that's at least what I understood from your reply) while lines B2040 and C2019 are not. Are there other, Wnt pathway related mutations in any of these lines? If so, this should be added to the results. Not everyone will want to spend the time to look up the genotype of these cells in previous publications while their genotype is rather important for the interpretation of the results presented here.

The authors performed the requested editorial changes.

***** Reviewer's comments *****

Referee #1 (Comments on Novelty/Model System for Author):

organoids were not generated for all models, so matching single cell observations with functional testing not possible for all

Referee #1 (Remarks for Author):

There have been significant improvements in clarity, data density and data presentation in the manuscript.
As a resource the manuscript is certainly worth publishing, and at minimum generates a well developped hypothesis.
A lot of effort hab been put into the manuscript for which we commend the authors.
We thank reviewer #1 for the positive comments.

However the findings remain hypothesis generating at best and cannot be considered validated or netirely accurate in it's present form. This however is possibly beyond the scope of this manuscript. If sufficient 'warning and context ' language is used, the manuscript can be correcltly used as a data rich basis for further validation of the concept.
As pointed out in the point by point reply page 5, the 6 clusters are possibly not the final CRC structure and applying them in a supervised way to external datasets does not constitute validation. Many other papers are published that stop at this level of validation indeed. What is more difficult are the conclusions relating MAPK gradients to the 6 clusters, if they are not stable then same for the rest...
Remarks of all revieuwers where in the same direction (see page 11 point by point section)and some of theselimitations should be highlighted.

We have added a dedicated paragraph to discuss these limitations of our study. See discussion, lines 372-378.

Last there is the point of what is tumorcell with CNV, without CNV or contaminating normal. From the point by point document it is clear that this is yet unresolved. An arguments is made that tumor cells may exist without genomic abnormalities and be paracrine driven. all fine, but the number of NVC number cells is quite high for some patients. However all downstream analysis used only CNV abberrant single cell data, removing all tumor biology of CNV stable cells in the disovery and conclusions. The bias that this introduces is unknown.
I suggest to highlight many of these possible biases in the manuscript. There are some well elaborated replies in the point by point document that aknowledge gaps , I suggest to incorporate these to drive also further work by the community in taking the next steps based on this work

We agree with reviewer #1 that analyses of the less prevalent CN-stable tumors (such as MSI)will require community efforts, as also for the question of how to distinguish between CN-normal tumor cells and CN-normal bystander epithelial cells. We have now outlined this limitation in lines 373 ff.

Referee #2 (Remarks for Author):

The authors have largely answered to my questions. There a re a few points which remain:

The authors have now added a part to the discussion that some of the TC1-4 cell phenotypes can be identified in CNN tumor cells and in normal adjacent tissue. However, the result text is still misleading regarding this interpretation of the data: it is still stated that "TC1-4 cells that have no normal tissue counterparts". This needs to be corrected.

The sentence was not meant to indicate “no TC1-4 transcriptomes were derived from normal tissue”, but merely “The TC1-4 clusters are not similar to known cell types defined for the normal colon cell hierarchy”. But we can see why the sentence is misleading and have thus rephrased the sentence (line 149)

It wasn't clear (or at least I couldn't find it) if the inhibitor treatments (BRAFi, MEKi, EGFRi) actually led to a block in organoid growth and massive cell death or just slowed down the cultures? Can you please add that information (e.g. survival curves) to the results?

In the time frame we observed in our study, growth is rather slowed down and there is no massive apoptosis. The data is already shown in Appendix Fig S6 (green cells: alive: apoptotic cells: red).

Since this wasn't clearly spelled out in the main text, please add to the results that OT227 and OT302 are mutant for APC (that's at least what I understood from your reply) while lines B2040 and C2019 are not. Are there other, Wnt pathway related mutations in any of these lines? If so, this should be added to the results. Not everyone will want to spend the time to look up the genotype of these cells in previous publications while their genotype is rather important for the interpretation of the results presented here.

We agree with the reviewer and have added our and previously published panel sequencing data for all organoid lines used into table EV2 (referenced from line 266).

2nd Revision - Editorial Decision 30th Jul 2021

We are pleased to inform you that your manuscript is accepted for publication and is now being se

EMBO PRESS

YOU MUST COMPLETE ALL CELLS WITH A PINK BACKGROUND ➔

PLEASE NOTE THAT THIS CHECKLIST WILL BE PUBLISHED ALONGSIDE YOUR PAPER

Corresponding Author Name: Markus Morkel, Nils Blüthgen
Journal Submitted to: EMBO Molecular Medicine
Manuscript Number: EMM-202114123

Reporting Checklist For Life Sciences Articles (Rev. June 2017)

This checklist is used to ensure good reporting standards and to improve the reproducibility of published results. These guidelines are consistent with the Principles and Guidelines for Reporting Preclinical Research issued by the NIH in 2014. Please follow the journal's authorship guidelines in preparing your manuscript.

A- Figures
1. Data
The data shown in figures should satisfy the following conditions:

- ➔ the data were obtained and processed according to the field's best practice and are presented to reflect the results of the experiments in an accurate and unbiased manner.
- ➔ figure panels include only data points, measurements or observations that can be compared to each other in a scientifically meaningful way.
- ➔ graphs include clearly labeled error bars for independent experiments and sample sizes. Unless justified, error bars should not be shown for technical replicates.
- ➔ if n< 5, the individual data points from each experiment should be plotted and any statistical test employed should be justified
- ➔ Source Data should be included to report the data underlying graphs. Please follow the guidelines set out in the author ship guidelines on Data Presentation.

2. Captions

Each figure caption should contain the following information, for each panel where they are relevant:

- ➔ a specification of the experimental system investigated (eg cell line, species name).
- ➔ the assay(s) and method(s) used to carry out the reported observations and measurements
- ➔ an explicit mention of the biological and chemical entity(ies) that are being measured.
- ➔ an explicit mention of the biological and chemical entity(ies) that are altered/varied/perturbed in a controlled manner.

- ➔ the exact sample size (n) for each experimental group/condition, given as a number, not a range;
- ➔ a description of the sample collection allowing the reader to understand whether the samples represent technical or biological replicates (including how many animals, litters, cultures, etc.).
- ➔ a statement of how many times the experiment shown was independently replicated in the laboratory.
- ➔ definitions of statistical methods and measures:
  - common tests, such as t-test (please specify whether paired vs. unpaired), simple $\chi 2$ tests, Wilcoxon and Mann-Whitney tests, can be unambiguously identified by name only, but more complex techniques should be described in the methods section;
  - are tests one-sided or two-sided?
  - are there adjustments for multiple comparisons?
  - exact statistical test results, e.g., P values = x but not P values < x;
  - definition of 'center values' as median or average;
  - definition of error bars as s.d. or s.e.m.

Any descriptions too long for the figure legend should be included in the methods section and/or with the source data.

In the pink boxes below, please ensure that the answers to the following questions are reported in the manuscript itself. Every question should be answered. If the question is not relevant to your research, please write NA (non applicable). We encourage you to include a specific subsection in the methods section for statistics, reagents, animal models and human subjects.

USEFUL LINKS FOR COMPLETING THIS FORM

http://www.antibodypedia.com
http://1degreebio.org
http://www.equator-network.org/reporting-guidelines/improving-bioscience-research-repor
http://grants.nih.gov/grants/olaw/olaw.htm
http://www.mrc.ac.uk/Ourresearch/Ethicsresearchguidance/Useofanimals/index.htm
http://ClinicalTrials.gov
http://www.consort-statement.org
http://www.consort-statement.org/checklists/view/32-consort/66-title
http://www.equator-network.org/reporting-guidelines/reporting-recommendations-for-tumo
http://datadryad.org
http://figshare.com
http://www.ncbi.nlm.nih.gov/gap
http://www.ebi.ac.uk/ega
http://biomodels.net/
http://biomodels.net/miriam/
http://jjj.biochem.sun.ac.za
http://oba.od.nih.gov/biosecurity/biosecurity_documents.html
http://www.selectagents.gov/

B- Statistics and general methods

Please fill out these boxes ➔ (Do not worry if you cannot see all your text once you press return)

1.a. How was the sample size chosen to ensure adequate power to detect a pre-specified effect size?	As the primary goal of this analysis was not to derive differences between patient groups, the sample size was chosen such that it represents a broad spectrum of the disease. Clustering of the data shows that the major cell clusters were not patient-specific.
1.b. For animal studies, include a statement about sample size estimate even if no statistical methods were used.	not applicable
2. Describe inclusion/exclusion criteria if samples or animals were excluded from the analysis. Were the criteria pre-established?	not applicable
3. Were any steps taken to minimize the effects of subjective bias when allocating animals/samples to treatment (e.g. randomization procedure)? If yes, please describe.	not applicable
For animal studies, include a statement about randomization even if no randomization was used.	not applicable
4.a. Were any steps taken to minimize the effects of subjective bias during group allocation or/and when assessing results (e.g. blinding of the investigator)? If yes please describe.	Organoid culture and analysis of organoid single cell data was performed by independent groups of researchers.
4.b. For animal studies, include a statement about blinding even if no blinding was done	not applicable
5. For every figure, are statistical tests justified as appropriate?	Yes. We used non-parametric tests throughout the manuscript, and used multiple-testing correction where necessary.
Do the data meet the assumptions of the tests (e.g., normal distribution)? Describe any methods used to assess it.	we used non-parametric tests throughout the study (Wilcox, Kruskal-Wallace) that do not rely on specific underlying distributions, but use ranks instead
Is there an estimate of variation within each group of data?	not applicable

Is the variance similar between the groups that are being statistically compared?	not applicable, as non-parametric tests insensitive to different variances were used

C- Reagents

6. To show that antibodies were profiled for use in the system under study (assay and species), provide a citation, catalog number and/or clone number, supplementary information or reference to an antibody validation profile. e.g., Antibodypedia (see link list at top right), 1DegreeBio (see link list at top right).	anti-TFF3 (Abcam; ab108599), anti-FABP (Abcam ab7366), anti-Epcam (ThermoScientific, MS144P1): supportive data by manufacturer and expected pattern in normal colon; anti-OLFM4 (Atlas, HPA077718), expected pattern in normal colon and supportive data in Antibodypedia; anti-Ki67 (Abcam, ab16667), anti-Lyz (Abcam, ab108508), KO-validated by manufacturer; anti-EREG (ThermoScientific, PA5-24727), anti-MUC2 (Leica, NCL-MUC-2, Anti-Ki67 (DAKO, M7240), anti-CK17 (Dako, M7046): validated for routine pathology at Charité University Medicine Berlin; CyTOF panel antibodies were individually validated in our labs, using individual combinations of cell lines and inhibitor treatments.
7. Identify the source of cell lines and report if they were recently authenticated (e.g., by STR profiling) and tested for mycoplasma contamination.	CRC organoid lines were generated as part of this study (P009T, P013T, NCO), previously generated in the study Schütte et al., 2018 (OT227, OT302) or bought from Hub Organoids (C2019, B2040). Identity of lines was validated by panel sequencing as in Materials and Methods. Organoid lines were regularly checked for mycoplasm contamination by commercial PCR test.

* for all hyperlinks, please see the table at the top right of the document

D- Animal Models

8. Report species, strain, gender, age of animals and genetic modification status where applicable. Please detail housing and husbandry conditions and the source of animals.	not applicable
9. For experiments involving live vertebrates, include a statement of compliance with ethical regulations and identify the committee(s) approving the experiments.	not applicable
10. We recommend consulting the ARRIVE guidelines (see link list at top right) (PLoS Biol. 8(6), e1000412, 2010) to ensure that other relevant aspects of animal studies are adequately reported. See author guidelines, under 'Reporting Guidelines'. See also: NIH (see link list at top right) and MRC (see link list at top right) recommendations. Please confirm compliance.	not applicable

E- Human Subjects

11. Identify the committee(s) approving the study protocol.	Ethics commission of Charité Universitätsmedizin Berlin
12. Include a statement confirming that informed consent was obtained from all subjects and that the experiments conformed to the principles set out in the WMA Declaration of Helsinki and the Department of Health and Human Services Belmont Report.	Informed consent was obtained from all patients used for single cell analysis and generation of organoids.
13. For publication of patient photos, include a statement confirming that consent to publish was obtained.	not applicable
14. Report any restrictions on the availability (and/or on the use) of human data or samples.	Human sequencing data is provided as count files, not nucleic acid sequences to protect the patients identity.
15. Report the clinical trial registration number (at ClinicalTrials.gov or equivalent), where applicable.	not applicable
16. For phase II and III randomized controlled trials, please refer to the CONSORT flow diagram (see link list at top right) and submit the CONSORT checklist (see link list at top right) with your submission. See author guidelines, under 'Reporting Guidelines'. Please confirm you have submitted this list.	not applicable
17. For tumor marker prognostic studies, we recommend that you follow the REMARK reporting guidelines (see link list at top right). See author guidelines, under 'Reporting Guidelines'. Please confirm you have followed these guidelines.	not applicable

F- Data Accessibility

18: Provide a "Data Availability" section at the end of the Materials & Methods, listing the accession codes for data generated in this study and deposited in a public database (e.g. RNA-Seq data: Gene Expression Omnibus GSE39462, Proteomics data: PRIDE PXD000208 etc.) Please refer to our author guidelines for 'Data Deposition'.<br><br>Data deposition in a public repository is mandatory for:<br>a. Protein, DNA and RNA sequences<br>b. Macromolecular structures<br>c. Crystallographic data for small molecules<br>d. Functional genomics data<br>e. Proteomics and molecular interactions	Scripts for processing of patient tissue scRNA sequencing data are available from https://github.com/molsysbio/sccrc. Scripts for processing of organoid RNA velocity data are available from https://github.com/molsysbio/sccrc_slamvelocity. Raw data is stored in GEO under the accession numbers GSE166555 (patient data) und GSE166556 (organoid data). Processed count data is available from https://sys-bio.net/sccrc.
19. Deposition is strongly recommended for any datasets that are central and integral to the study; please consider the journal's data policy. If no structured public repository exists for a given data type, we encourage the provision of datasets in the manuscript as a Supplementary Document (see author guidelines under 'Expanded View' or in unstructured repositories such as Dryad (see link list at top right) or Figshare (see link list at top right).	not applicable
20. Access to human clinical and genomic datasets should be provided with as few restrictions as possible while respecting ethical obligations to the patients and relevant medical and legal issues. If practically possible and compatible with the individual consent agreement used in the study, such data should be deposited in one of the major public access-controlled repositories such as dbGAP (see link list at top right) or EGA (see link list at top right).	Clinical characteristis of patients are given as table S1. Human sequencing data is provided as count files, not nucleic acid sequences to protect the patients identity.
21. Computational models that are central and integral to a study should be shared without restrictions and provided in a machine-readable form. The relevant accession numbers or links should be provided. When possible, standardized format (SBML, CellML) should be used instead of scripts (e.g. MATLAB). Authors are strongly encouraged to follow the MIRIAM guidelines (see link list at top right) and deposit their model in a public database such as Biomodels (see link list at top right) or JWS Online (see link list at top right). If computer source code is provided with the paper, it should be deposited in a public repository or included in supplementary information.	code assiciated with this study is deposited under sys-bio.net/sccrc/.

G- Dual use research of concern

22. Could your study fall under dual use research restrictions? Please check biosecurity documents (see link list at top right) and list of select agents and toxins (APHIS/CDC) (see link list at top right). According to our biosecurity guidelines, provide a statement only if it could.	not applicable
